# 2-Desaza-annomontine (C81) impedes angiogenesis through reduced VEGFR2 expression derived from inhibition of CDC2-like kinases

**DOI:** 10.1007/s10456-024-09906-y

**Published:** 2024-02-26

**Authors:** T. J. Zech, A. Wolf, M. Hector, I. Bischoff-Kont, G. M. Krishnathas, S. Kuntschar, T. Schmid, F. Bracher, T. Langmann, R. Fürst

**Affiliations:** 1 Faculty of Biochemistry, Chemistry and Pharmacy, Institute for Pharmaceutical Biology, Goethe University Frankfurt, Frankfurt, Germany; 2 Laboratory for Experimental Immunology of the Eye, Department of Ophthalmology, Faculty of Medicine and University Hospital Cologne, University of Cologne, Cologne, Germany; 3 Centre for Molecular Medicine Cologne (CMMC), University of Cologne, Cologne, Germany; 4 Faculty of Medicine, Institute of Biochemistry I, Goethe University Frankfurt, Frankfurt, Germany; 5 Pharmaceutical Chemistry, Department of Pharmacy, Center for Drug Research, Ludwig-Maximilians-Universität München, Munich, Germany; 6 Pharmaceutical Biology, Department of Pharmacy, Center for Drug Research, Ludwig-Maximilians-Universität München, Munich, Germany

**Keywords:** Angiogenesis, CDC-2-like-kinases, Kinase inhibitor, Splicing, WNT-signaling, Natural product

## Abstract

Angiogenesis is a crucial process in the progression of various pathologies, like solid tumors, wet age-related macular degeneration, and chronic inflammation. Current anti-angiogenic treatments still have major drawbacks like limited efficacy in diseases that also rely on inflammation. Therefore, new anti-angiogenic approaches are sorely needed, and simultaneous inhibition of angiogenesis and inflammation is desirable. Here, we show that 2-desaza-annomontine (C81), a derivative of the plant alkaloid annomontine previously shown to inhibit endothelial inflammation, impedes angiogenesis by inhibiting CDC2-like kinases (CLKs) and WNT/*β*-catenin signaling. C81 reduced choroidal neovascularization in a laser-induced murine in vivo model, inhibited sprouting from vascular endothelial growth factor A (VEGF-A)-activated murine aortic rings ex vivo, and reduced angiogenesis-related activities of endothelial cells in multiple functional assays. This was largely phenocopied by CLK inhibitors and knockdowns, but not by inhibitors of the other known targets of C81. Mechanistically, CLK inhibition reduced VEGF receptor 2 (VEGFR2) mRNA and protein expression as well as downstream signaling. This was partly caused by a reduction of WNT/*β*-catenin pathway activity, as activating the pathway induced, while *β*-catenin knockdown impeded VEGFR2 expression. Surprisingly, alternative splicing of VEGFR2 was not detected. In summary, C81 and other CLK inhibitors could be promising compounds in the treatment of diseases that depend on angiogenesis and inflammation due to their impairment of both processes.

**Graphical abstract:**

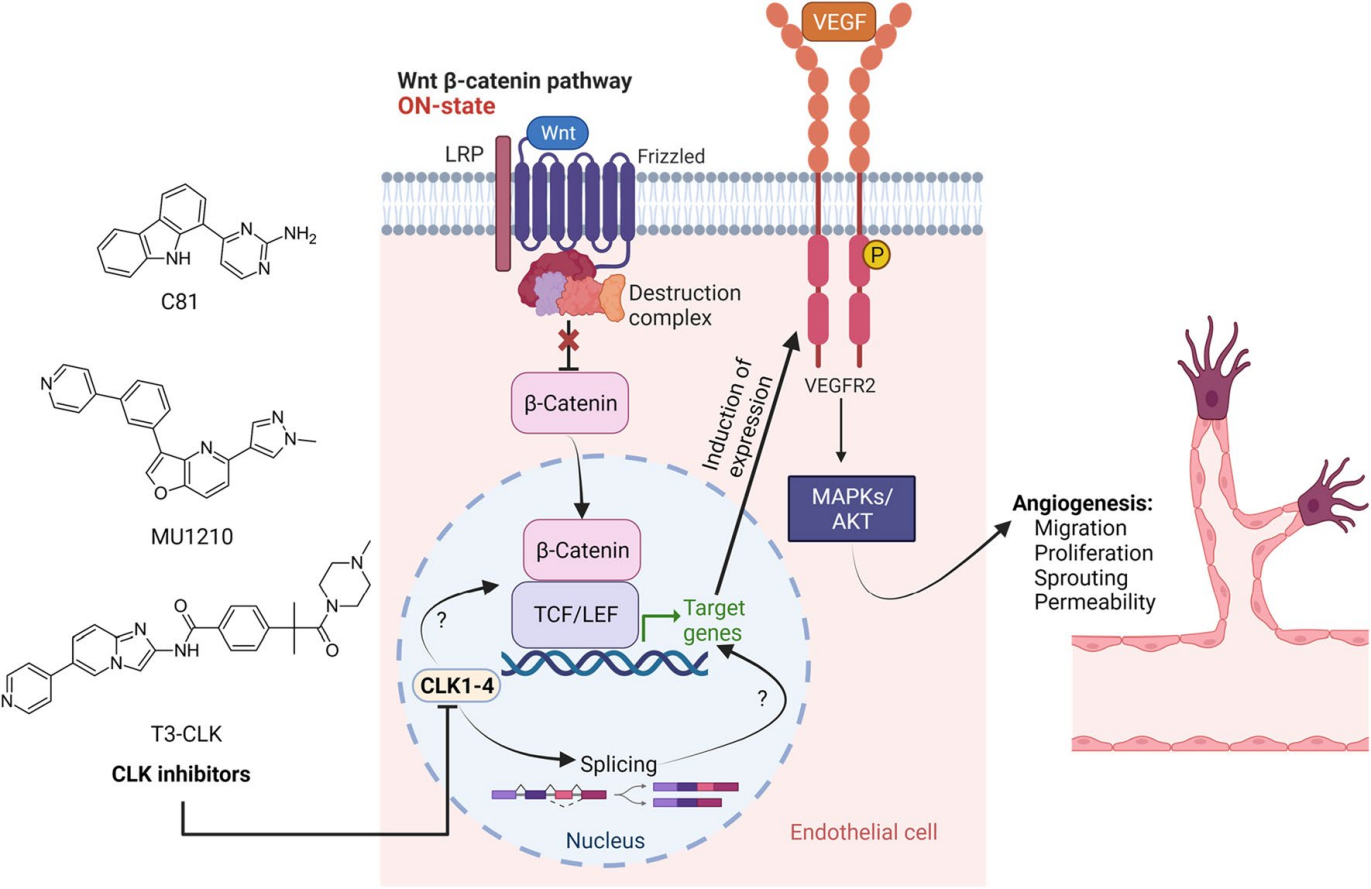

**Supplementary Information:**

The online version contains supplementary material available at 10.1007/s10456-024-09906-y.

## Introduction

The formation of blood vessels from pre-existing ones, called angiogenesis, is an essential physiological process, especially in growth and wound healing [1, 2]. It is a complex, multi-step process that is often initiated by a lack of oxygen, which leads to a release of pro-angiogenic growth factors, like vascular endothelial growth factor A (VEGF-A, henceforth called VEGF), causing a pro-angiogenic shift in the otherwise carefully kept equilibrium of pro- and anti-angiogenic factors [2, 3]. VEGF then binds to its main activating receptor in the vascular endothelium, VEGFR2, and initiates downstream signaling cascades, e.g., the mitogen-activated protein kinase (MAPK) or protein kinase B (PKB/AKT) pathways [2, 4]. This causes the blood vessels to dilate, increases permeability, and leads endothelial cells to form nascent sprouts into the surrounding tissue, which elongate through migration and proliferation of two distinct endothelial cell phenotypes, called tip and stalk cells [2]. Finally, a new blood vessel is formed when two sprouts connect [2]. However, in various pathophysiological conditions, this tightly controlled process can become dysregulated and cause aberrant growth of leaky and only partially functional vessels, caused by hyperstimulation of the endothelium [5–7]. Subsequently, this can contribute to the progression of diseases, like cancer, wet age-related macular degeneration (wAMD), and chronic inflammatory diseases, like arthritis [8].

Therefore, anti-angiogenic strategies have been introduced in the pharmacotherapy of solid tumors and wAMD with some success [7, 9]. However, the efficacy of these treatments is still limited, partly because underlying inflammatory processes still contribute to the diseases [10], but also because of resistance mechanisms that allow diseases to induce angiogenesis despite anti-angiogenic treatment [2]. In inflammatory diseases, inhibition of angiogenesis is still not a commonly used therapeutic approach. Accordingly, the search for anti-angiogenic compounds is still an ongoing endeavor, and compounds with combined effects on angiogenic and inflammatory processes could provide a benefit in the treatment of diseases that depend on both.

Over almost the entire history of medicine, natural compounds have been an important source in the search for new treatment options, originally in traditional medicine, but also nowadays, as they often present interesting and diverse structural features, targets, and mechanisms of action [11]. We have previously characterized 2-desaza-annomontine (C81), a derivative of the plant alkaloid annomontine [12], as a potent modulator of endothelial inflammatory functions, and an inhibitor of the kinases dual-specificity regulated kinase 2 (DYRK2), Pim3 proto-oncogene (PIM3), and the CDC2-like kinases CLK1 and CLK4 [13]. Of these kinases, especially CLKs have recently been gaining interest as promising targets in the treatment of solid tumors and inflammatory diseases, like osteoarthritis, which are very closely associated with pathological angiogenesis [2, 8, 14–17]. CLKs are a family of four highly conserved dual-specificity kinases that are best known for their role in the phosphorylation of serine/arginine-rich splicing factors (p-SR proteins), a crucial event for exon recognition and spliceosome assembly in the splicing process [15]. Some CLK inhibitors, i.e., lorecivivint and cirtuvivint, have even made it into clinical trials for the treatment of the diseases mentioned above, with some success in the pre- and early clinical stages [14, 17–19]. This success is generally ascribed to their potency as inhibitors of the WNT/*β*-catenin signaling cascade [14, 18], a pathway that has been shown to influence a large number of cellular processes, including angiogenic function in some types of endothelial cells [20, 21]. Interestingly, the effect of these compounds, or CLK inhibition in general, on angiogenic function in the endothelium has not been explored to date. Therefore, we chose to apply C81, with its known in vivo activity against inflammatory processes in the endothelium [13], to establish whether it inhibits angiogenic cell functions as well. Additionally, we set out to investigate the underlying mechanism of action to uncover whether CLKs are a useful target in the treatment of angiogenesis-related diseases and, if so, through which mechanisms they affect endothelial angiogenic functions.

We achieved this using C81 in state-of-the-art in vivo and ex vivo models for angiogenesis, namely a murine laser-induced choroidal neovascularization model and a murine aortic ring assay. Additionally, we investigated angiogenesis-related cell functions like migration, proliferation, and sprouting of human umbilical vein endothelial cells (HUVECs). Subsequently, we sought to uncover the underlying pharmacological mechanism of action of C81 using selective chemical probes and knockdown experiments for the kinases targeted by C81.

## Materials and methods

### Compounds

C81 was kindly provided by the group of Prof. Franz Bracher (Pharmaceutical and Medicinal Chemistry, Department of Pharmacy – Center for Drug Research, Ludwig-Maximilians-Universität München, Munich, Germany) with a purity of 98.4% [12, 13]. The CLK inhibitors MU1210 and T3-CLK were purchased from Sigma-Aldrich (St. Louis, Missouri, USA) or were kindly provided by the group of Prof. Stefan Knapp (SGC, Institute of Pharmaceutical Chemistry, Goethe University Frankfurt, Frankfurt, Germany). The DYRK2 inhibitor LDN-192,960 and the glycogen synthase kinase-3 (GSK3) inhibitor LY2090314 were purchased from MedChemExpress (Monmouth Junction, New Jersey, USA); the PIM kinase inhibitor AZD1208 was obtained from Selleckchem (Houston, Texas, USA). The chemical structures of C81, MU1210, and T3-CLK can be found in Supp. Figure 1. All kinase inhibitors, including C81, were dissolved and aliquoted in DMSO (Sigma-Aldrich) at concentrations ranging from 10 to 100 mM, adjusted for solubility to achieve the lowest possible DMSO concentrations in the final cell culture experiments and stored at − 80 °C until use. In cell culture and ex vivo experiments, DMSO concentrations never exceeded 0.1%, whereas in in vivo animal experiments, DMSO concentrations never exceeded 0.01% in the eye.

### Animals

All in vivo animal procedures were conducted in compliance with protocols approved by the local governmental authorities (Tierschutzkommission acc. § 15 TSchG of the Landesamt für Natur, Umwelt und Verbraucherschutz Nordrhein-Westfalen, with the permission number Az 81-02.04.2022.A338) and were in accordance with the National Institutes of Health (NIH Bethesda, Maryland, USA) guidelines. Mice were housed in individually ventilated caging (IVC) systems (GM 500, Tecniplast Greenline) with a maximum cage density of five adult mice per cage. Light was adjusted to a 12-h/12-h light/dark cycle with light on at 6 am, temperature and relative humidity were regulated to 22 ± 2 °C, and 45–65% relative humidity. Mice were fed irradiated phytoestrogen-free standard diet for rodents (Altromin 1314; 59% carbohydrates, 27% protein, 14% fat) and had access to food and acidified water ad libitum. 8- to 10-week-old C57BL/6J mice with an averaged body weight of 19 g ± 1.5 g for females and 25 g ± 2 g for males were used for experiments.

Ex vivo animal procedures were performed in compliance with the German Animal Welfare Act (§ 4 Tierschutzgesetz) and approval number V54-19c20/21I-FR/Biologicum, Tierhaus Campus Riedberg (Regierungspräsidium Darmstadt, Germany). The ex vivo animal experiments were conducted using 4- to 6-week-old C57BL/6 N mice, which were kept under a 12-h/12-h light/dark cycle and had access to food and water *ad libitum* until sacrifice.

### Laser photocoagulation

Laser photocoagulation was carried out as described previously [22]. In brief, mice were anesthetized with a mixture of ketamine (100 mg/kg body weight, Ketavet) and xylazine (5 mg/kg body weight, 2% Rompun) diluted in 0.9% sodium chloride by intraperitoneal (i.p.) injection. Their pupils were dilated with a topical drop of phenylephrine 2.5%/tropicamide 0.5%. A slit lamp-mounted diode laser system (Quantel Medical Vitra, 532-nm green laser, power 100 mW, duration 100 ms, and spot size 100 μm) was used to generate three equal laser burns around the optic nerve in each eye with a cover glass as a contact lens. To validate rupture of Bruch’s membrane, infrared (IR) images were recorded using Spectralis HRA/OCT device to analyze post-laser retinal structure and laser lesion size in vivo. Exclusion criteria were cataract and corneal epithelial edema before laser photocoagulation, unsuccessful laser burns without Bruch’s membrane rupture, or severe choroidal hemorrhages.

### Intravitreal drug administration

The animals were randomly assigned to the experimental groups. The following compounds (all diluted in phosphate-buffered saline, PBS) were injected intravitreally immediately after laser pulse application. 1 µl of either 15-µM or 50-µM C81 or corresponding vehicle controls (0.015% or 0.05% DMSO) were applied to reach 3 µM or 10 µM final concentration assuming 4-µl vitreous volume [23]. Therefore, eyes were treated with oxybuprocaine (Conjuncain, 0.4 mg/ml) eye drops and a 34-gauge needle was inserted into the vitreous space approximately 1.5 mm below the limbus and the compounds were administered bilaterally with a NanoFil syringe (Word Precision Instruments, Sarasota, FL, USA). Afterward, eyes were covered with Bepanthen eye and nose ointment (Bayer, Leverkusen, Germany).

### Fundus photography and fundus fluorescein angiography (FFA)

Vascular leakage was analyzed 3 and 7 days after laser photocoagulation. After anesthesia and pupil dilatation, mice received i.p. injection of 0.1 ml of 2.5% fluorescein diluted in 0.9% sodium chloride. Late-phase angiograms were recorded 10 min after fluorescein injection using Spectralis HRA/OCT. Simultaneously, IR fundus images were acquired to analyze the laser lesion size. The size of laser lesions and vascular leakage was determined using the measuring tool of the HEYEX software (Heidelberg Engineering, Heidelberg, Germany). The analysis of vascular leakage by measuring pixel intensities was performed as described previously [24].

### Immunohistochemistry of retinal and RPE/choroidal flat mounts

Mice were euthanized by cervical dislocation and the eyes enucleated and fixed in 4% of Roti-Histofix (Carl Roth, Karlsruhe, Germany) for 3 h at room temperature (RT). The dissected RPE/choroidal flat mounts were permeabilized and blocked overnight in Perm/Block buffer (5% normal donkey serum (NDS), 0.2% bovine serum albumin (BSA), 0.3% Triton X-100 in PBS) at 4 °C. RPE/choroidal flat mounts were stained in addition with FITC-conjugated isolectin B4 from *Bandeiraea simplicifolia* (BS, 1:100 diluted in Perm/Block, L2895, Sigma-Aldrich). After several washing steps, retinal and RPE/choroidal flat mounts were mounted on a microscope slide and embedded with fluorescence mounting medium (Vectashield HardSet H-1400, Vector Labs, Newark, California, USA). Images were taken with a Zeiss Imager. M2 equipped with an ApoTome.2 (Carl Zeiss, Oberkochen, Germany). Areas of CNV in RPE/choroidal flat mounts were measured with the spline function of the graphic tool included in the ZEN software (Zeiss). The average CNV area per eye was calculated.

### Ex vivo mouse aortic ring assay

Mouse aortic ring assays were performed with 4- to 6-week-old C57BL6/N mice, which were kindly provided by the group of Prof. Achim Schmidtko (Institute of Pharmacology and Clinical Pharmacy, Goethe University Frankfurt, Frankfurt, Germany), as previously described [25, 26]. Briefly, mice were sacrificed using CO_2_. Death was ensured by subsequent breaking of the necks. Aortae were explanted, cleaned of surrounding tissue, and cut into rings of about 0.5–1 mm length. These rings were then incubated overnight in Opti-MEM I (Gibco/Thermo Scientific, Waltham, Massachusetts, USA) supplemented with 100-U/ml penicillin and 100-µg/ml streptomycin (P/S; Pan-Biotech, Aidenbach, Germany). On the following day, rings were embedded into a 50-µl rat tail collagen I gel (1.5 mg/ml in M199; Corning, Corning, New York, USA) and incubated with 150 µl of Opti-MEM I supplemented with P/S, 2.5% FCS Superior (Sigma-Aldrich), and 30-µg/ml murine vascular endothelial growth factor (mVEGF_165_; PeproTech, Rocky Hill, New Jersey, USA) until first endothelial sprouts were visible (3–5 days). Afterward, sprouting rings were treated with 3-µM C81 or vehicle control for 3 additional days. Subsequently, the stimulation was terminated by fixating the rings with ROTI-Histofix (Carl Roth) for 30 min. To stain the rings, they were first permeabilized using 0.25% Triton X-100 (Carl Roth) twice at room temperature. Unspecific binding sites were blocked using 1% BSA (Carl Roth) in PBS at 4 °C overnight, followed by staining with FITC-coupled BS-I Lectin (L9381, 0.1 mg/ml, Sigma-Aldrich) and a CY3-conjugated antibody against smooth muscle actin (α-SMA, C6198, Dilution 1:1000, Sigma-Aldrich) at 4 °C overnight. After thorough washing of the rings with 0.1% Triton X-100, images were taken using a confocal laser scanning microscope (LSM 780, Zeiss), and sprouting was quantified manually using Fiji/ImageJ (version 1.53t, NIH).

### Cell culture

HUVECs were bought from PELOBiotech (Martinsried, Germany) or were isolated from human umbilical cords of anonymized, healthy donors as previously described by Jaffe et al. [27] (the research Ethics Committee/Institutional Review Board approved the waiver W1/21Fü for the use of anonymized human material on September 15th, 2021). Briefly, the umbilical veins were washed with warm PBS including Ca^2+^ and Mg^2+^ (PBS+) to remove remaining cord blood and then incubated with a collagenase A solution (0.1 g/l; Roche, Basel, Switzerland) for 45 min at 37 °C. Afterward, cells were detached from the vessel walls by gentle tapping on the outside of the umbilical cords, flushed out using warm M199 (PAN-Biotech) supplemented with 10% FCS (Sigma-Aldrich) and P/S (PAN-Biotech) and collected. Subsequently, the cells were pelleted by centrifugation at 300 g for 5 min, the supernatant was discarded, and cells were resuspended in endothelial cell basal medium (PELOBiotech) containing 10% FCS (Sigma-Aldrich), P/S (PAN-Biotech), 2.5-µg/ml amphotericin B (PAN-Biotech), and EASY endothelial cell growth supplement (PELOBiotech). This medium will henceforth be called fully supplemented ECGM. Cell suspensions were then seeded on 25 cm^2^ cell culture flasks (Sarstedt, Nümbrecht, Germany) coated with collagen G (Sigma-Aldrich). HUVECs were generally split every 2–4 days at a ratio of 1:3, expanded until passage 2 and, for experimental purposes, exclusively used in passage 3. HMEC-1, a microvascular cell line [28], were obtained from the CDC (lot 119223; Centers for Disease Control and Prevention, Atlanta, Georgia, USA) and cultivated on collagen G-coated 25 cm^2^ or 75 cm^2^ flasks (Sarstedt) using fully supplemented ECGM (PELOBiotech). They were split every 2–3 days at a ratio of 1:3 and used up to passage 30. Stably transfected HMEC-1 were only used until passage 15.

### Spheroid sprouting assay

HUVECs or HMEC-1 spheroids were generated using the hanging drop method, as previously described [29]. Briefly, 400 HUVECs or HMEC-1 were seeded as droplets onto square petri dishes (Greiner Bio-One, Kremsmünster, Austria), which were flipped upside down and incubated for 24 h. Spheroids were then collected from the droplets by flushing with PBS+, washed, and then embedded in a rat tail collagen I gel (1.5 mg/ml in M199, Corning) containing methylcellulose (Sigma-Aldrich) and 5% FCS (Sigma-Aldrich). After collagen polymerization, the spheroids were treated with the indicated compounds at the indicated concentrations for 30 min. Subsequently, sprouting was induced using human recombinant VEGF_165_ (PeproTech) at 10 ng/ml for 20 h, after which spheroids were fixed using ROTI-Histofix (Carl Roth) for 30 min. Finally, images were taken using a Leica DMI IL LED inverted microscope (Leica Microsystems, Wetzlar, Germany) and analyzed manually using Fiji/ImageJ (NIH).

### Proliferation assay

1,500 cells (HUVECs or HMEC-1) per well were seeded on collagen G-coated 96-well plates (Greiner Bio-One) and grown in fully supplemented ECGM (PELOBiotech) for 24 h. Afterward, cells were either washed, fixated using methanol/ethanol (2:1) for 10 min, and then stained using crystal violet (Carl Roth), or treated with the indicated concentrations of C81 or vehicle control for another 72 h. The incubation of the treated cells was then stopped as described above. After staining with crystal violet, cells were washed using water until the water ran clear. Crystal violet was leached using 20% acetic acid, and absorbance was measured using a plate reader (Tecan, Männedorf, Switzerland). Cells fixated after 24 h were used for baseline normalization.

### Scratch assay

Undirected migration was studied using a scratch assay. For this, cells (HUVECs or HMEC-1) were seeded on 24-well plates (Greiner Bio-One) and grown to confluency. Consequently, a scratch was introduced to the monolayer using a 10-µl XL pipette tip (Greiner Bio-One), after which cells were washed and treated with the indicated concentrations of the indicated compounds, a vehicle control, and a serum starvation control (1% FCS in M199). Cells were allowed to migrate for 9 to 12 h or until the scratches in the vehicle control were mostly closed. Subsequently, images were taken using a DMI IL LED inverted microscope (Leica Microsystems) and quantified using Fiji/ImageJ (NIH). Serum starvation controls served as a baseline.

### Boyden chamber assay

100,000 cells (HUVECs) were seeded on collagen G-coated cell culture inserts (Corning) made from polycarbonate with a pore size of 8 μm and left to adhere for 2–3 h. Subsequently, cells were treated with the indicated concentrations of C81, introduced to a 0–20% FCS gradient (0% FCS in the upper chamber, 20% in the lower chamber) in M199, and allowed to migrate for 16 h. Afterward, the cells were washed with PBS, fixated using methanol/ethanol (2:1), stained with crystal violet, and then washed again until PBS ran clear. Cells that did not migrate were removed from the top of the insert by gently scraping them off with a cotton swab. Finally, remaining crystal violet was leached using 20% acetic acid, and absorbance was measured using a plate reader (Tecan).

### Live-cell chemotaxis assay

2D chemotaxis of scarcely seeded HUVECs was evaluated using µ-Slide Chemotaxis slides (ibidi, Martinsried, Germany) according to the manufacturer’s instructions. For this, 18,000 HUVECs were seeded onto the channel of the slide and left to adhere for 2 h. Afterward, cells were washed with M199 (Sigma-Aldrich) containing P/S (PAN-Biotech), treated with 10-µM C81 or vehicle control and subjected to an FCS (Sigma-Aldrich) gradient (0–20%). Subsequently, cells were allowed to migrate for 20 h in a climate chamber (5% CO_2_, 37 °C), and microscopic images were taken every 10 min on a DMI6000 B microscope (Leica Microsystems). Migration was analyzed using the Manual Tracking and Chemotaxis Analysis plugins for Fiji/ImageJ (NIH).

### Tube formation assay

Tube formation was measured using a Matrigel (Corning) based assay. Initially, wells in a µ-slides Angiogenesis (ibidi) were coated with 10-µl growth factor-reduced Matrigel (Corning). Subsequently, 10,000 HUVECs per well were seeded onto the Matrigel in fully supplemented ECGM containing the indicated treatments. HUVECs were allowed to form tubes for 7.5 h, after which images taken immediately using a Leica DMI6000 B inverted microscope. Images were then quantified for number of junctions and number of master segments using the angiogenesis analyzer plugin for Fiji/ImageJ (NIH).

### SDS-PAGE and western blot analysis

HUVECs or HMEC-1 were grown to confluency and treated as indicated. Unless specified otherwise, treatments were performed in fully supplemented ECGM. After the incubation times were over, cells were washed with cold PBS and lysed using a RIPA buffer containing PMSF (Roche), NaF (Sigma-Aldrich), EDTA-free Complete Mini (Roche), and Na_3_VO_4_ (Sigma-Aldrich). If phosphoproteins were analyzed, the lysis buffer additionally contained phosphatase inhibitors. Afterward, the protein concentrations of the lysates were determined using a bicinchoninic acid assay kit (Thermo Scientific) according to the manufacturer’s instructions. Subsequently, a Tris–HCl (Carl Roth)-based buffer containing pyronin Y (Sigma-Aldrich), sodium dodecyl sulfate (SDS, Sigma-Aldrich), glycerol (Carl Roth), and dithiothreitol (DTT, Sigma-Aldrich) were added to each sample, and proteins were denatured by heating the samples to 95 °C for 5 min. Consequently, 20–25 µg of protein was run on a polyacrylamide gel (between 7.5 and 15%, depending on the analyzed proteins, Carl Roth) and blotted onto a 0.2-μm polyvinylidene fluoride (PVDF) membrane (Bio-Rad, Hercules, California, USA) using a Transblot Turbo device (Bio-Rad) or by tank blotting at 30 V for 16 h or 100 V for 1 h. Unspecific binding sites were then blocked using BSA (Carl Roth) or non-fat dried milk (Blotto, Carl Roth), both at 5% in TBS containing 0.1% Tween 20. Finally, the membranes were incubated with antibodies for 2 h at room temperature (RT) or overnight at 4 °C, both conditions with gentle shaking, and visualized using luminol-based enhanced chemiluminescence (ECL) and X-ray films (Fujifilm, Tokyo, Japan) or a ChemiDoc XRS+ (Bio-Rad) imager. The images were quantified using the densitometry feature of Fiji/ImageJ (NIH). When two or more proteins of similar sizes were analyzed on the same membrane, previous antibodies were stripped off the membrane by incubating the membrane for 20 min using an acidic (pH 2.2) stripping buffer containing glycine (Carl Roth), 0.1% SDS (Sigma-Aldrich), and 1% Tween 20 (Carl Roth). Afterward, membranes were washed, and unspecific binding sites were blocked again before membranes were incubated with the next antibody. The following primary antibodies were used: anti-VEGF receptor 2 (55B11) rabbit mAb #2479 (dilution 1:2000; Cell Signaling Technology, CST, Danvers, Massachusetts, USA); anti-phospho-p44/42 MAPK (Erk1/2) (Thr202/Tyr204) (E10) mouse mAb #9106 (dilution 1:2000; CST); anti-p44/42 MAPK (Erk1/2) antibody #9102 (dilution 1:1000; CST); anti-phospho-AKT (Ser473) rabbit mAb #4060 (dilution 1:2000, CST); anti-AKT (pan) rabbit mAb #4691 (dilution 1:2000, CST); mouse monoclonal anti-*β*-actin peroxidase-linked antibody A3854 (dilution 1:50.000, Sigma-Aldrich); mouse anti-phospho-epitope SR proteins antibody, clone 1H4 MABE50 (dilution 1:1000; Sigma-Aldrich); and anti-*β*-catenin (D10A8) XP rabbit mAb #8480 (dilution 1:2000; CST). Secondary antibodies were goat anti-rabbit IgG, HRP-linked antibody #7074 (dilution 1:3000; CST) and horse anti-mouse IgG, HRP-linked antibody #7076 (dilution 1:3000; CST).

### Quantitative real-time PCR (qPCR)

To assess relative mRNA expression, quantitative real-time PCR was performed. For this, cells were initially subjected to the indicated conditions, after which total RNA was isolated using the RNeasy Mini Kit (Qiagen, Hilden, Germany) according to the manufacturer’s instructions, including on-column DNAse digestion. 1 µg of isolated RNA was then reverse transcribed into cDNA using FastGene Scriptase II (Nippon Genetics Europe, Düren, Germany) with random hexamer primers (New England Biolabs NEB, Ipswich, Massachusetts, USA) according to the manufacturer’s protocol. cDNA was then diluted 1:25, and gene expression was quantified using the primers stated in Table 1 and the SyGreen Blue Hi-ROX Mastermix (PCR Biosystems, London, United Kingdom) on a StepOne Plus device (Applied Biosystems/Thermo Scientific). Relative Gene expression was quantified with the ΔΔCt method using GAPDH as the control gene.

**Table 1:** Primers used for qPCR

Primer name	Sequence (5′-3′)
GAPDH forward	CCACATCGCTCAGACACCAT
GAPDH reverse	TGAAGGGGTCATTGATGGCAA
VEGFR2 forward	GTGACCAACATGGAGTCGTGT
VEGFR2 reverse	AGCTGATCATGTAGCTGGGAA
CLK1 forward	AGCAAACACAGGATTCACCAC
CLK1 reverse	CGTCTCCACTCTGACAGATCA
CLK2 forward	GAGCCGAAAGCATAAGCGAC
CLK2 reverse	TCCCCGATCCCGGCTATAAT
CLK3 forward	AGGTCCTACAGTCGGGAACA
CLK3 reverse	CGACGATGACGAGAACGTGA
CLK4 forward	ATTTTGTGGGGTGTTTGTCGC
CLK4 reverse	GCTTTCATGTCCCCAGCTTTC
TCF7L2 forward	CATCCGGCCATAGTCACACC
TCF7L2 reverse	AACGTGCACTCAGCTACGAC
MYC forward	CGTCCTCGGATTCTCTGCTC
MYC reverse	GCTGGTGCATTTTCGGTTGT
DVL1 forward	AACAAGATCACCTTCTCCGAG
DVL1 reverse	ACTGGAGCCACTGTTGAGGT
TCF7 forward	CCTGCGGACATCAGCCAGAA
TCF7 reverse	TCAGGGAGTAGAAGCCAGAGAG

### Kinase assay

Affinity for C81 against CLKs was measured by Eurofins DiscoveRx (San Diego, California, USA) using their KdELECT Assay Platform in 11 concentrations ranging from 0.1 to 10 µM in duplicates.

### Assessment of interchromatin granule clusters (IGCs)

55,000 HUVECs were grown on collagen G-coated 8-well slides with coverslip bottoms (ibidi) until confluency and then incubated with the indicated concentrations of the compounds for 6 h. Afterward, cells were washed with cold PBS and fixated using ROTI-Histofix (Carl Roth) for 10 min. Next, the fixated HUVECs were permeabilized using 0.1% Triton X-100 (Carl Roth), and unspecific binding sites were blocked using 1% BSA (Carl Roth) in PBS. Subsequently, the same antibody against phosphorylated SR proteins as used for western blotting (MABE50, dilution 1:500; Sigma-Aldrich) was applied to stain IGCs and visualized using a secondary anti-mouse antibody coupled to Alexa 488 (dilution 1:400; Thermo Scientific). Hoechst 33342 (Sigma-Aldrich) served as a control stain of the nuclei. Images were taken using a Leica DMI6000 B epifluorescence microscope (Leica Microsystems).

### siRNA transfection and knockdowns

HUVECs were transfected with siRNAs targeting CLK1, CLK2, CLK3, CLK4, *β*-catenin (CTNNB1) or a non-targeting control (all Dharmacon ON-TARGET Plus SMARTpools, Horizon Discovery, Lafayette, Colorado, USA) using either Lipofectamine RNAiMAX (Thermo Scientific) or GeneTrans II (MoBiTec, Goettingen, Germany) according to the manufacturer’s instructions. When Lipofectamine RNAiMAX was used, HUVECs were transfected with 25-pmol siRNA per well in a 6-well plate for 24 h and afterward incubated or treated as indicated. When Genetrans II was used, HUVECs were transfected with 80-pmol siRNA per well in a 6-well plate, prediluted in Diluent B, for 4 h, and afterward treated as indicated. Unless otherwise specified, incubation timepoints are counted from the beginning of the transfection. Analysis was performed using the previously described spheroid sprouting assay, SDS-PAGE with subsequent western blot analysis, or qPCR.

### Propidium iodide staining and flow cytometry

Apoptosis was measured using the method described by Nicoletti et al. [30]. Briefly, HUVECs were treated with the indicated concentrations of MU1210, staurosporine (positive control), or vehicle control for 24 h. Afterward, cells were washed and detached from the culture vessels; supernatants and all washing solutions were conserved. Subsequently, the cells were centrifuged at 300×*g* and 4 °C for 10 min and stained using a hypotonic fluorochrome solution (HFS) containing Triton X-100 (Carl Roth) and propidium iodide (Carl Roth) for 24 h at 2–8 °C. Consequently, the fluorescence intensity of single cells was analyzed using a flow cytometer (FACSVerse, BD, Franklin Lakes, New Jersey, USA). Doublets were removed through gating; lower fluorescence intensity than typical for G0/G1 phase cells indicated DNA degradation associated with apoptosis. Alternatively, propidium iodide staining was used to determine cell cycle distribution of proliferating HUVECs. When this was done, 44,000 HUVECs were seeded in a well of a 6-well plate in fully supplemented ECGM and left untreated for 24 h, after which they were treated as indicated. 48 h later, cells were washed, detached, stained, and measured as described above. Cell cycle distribution was determined from the fluorescence intensity of the cells.

### RNA sequencing (RNA-Seq)

To generate hypotheses about potentially affected pathways as well as alternative splicing events, RNA-Seq was performed. For this, confluent HUVECs were treated with 10-µM C81, MU1210, or a vehicle control for 6 h, after which total RNA was isolated using the RNeasy Micro Kit (Qiagen) according to the manufacturer’s instructions. The quality of the RNA was then assessed using a Tapestation 4150 (Agilent, Santa Clara, California, USA), and libraries were prepared using the Lexogen Corall Total RNA-Seq Kit (Lexogen, Vienna, Austria) with the poly-A selection module and the qPCR module according to the manufacturer’s instructions. The quality of the libraries was verified using a Tapestation 4150 (Agilent) and quantified using a Qubit 3 Fluorometer (Thermo Scientific). Libraries were then sequenced on a NextSeq 2000 Device (Illumina, San Diego, California, USA) using paired end reads of 105 bp length and approximately 50 million reads per sample. Post-processing of reads (quality control, trimming, alignment) was done using the BlueBee platform included with the library prep kit.

### Gene ontology (GO) term analysis

Differentially expressed genes were detected from the RNA-Seq data using DESeq2 (Version 1.36.0) for R (version 4.2.1) [31]. Subsequently, downregulated genes, as defined by an adjusted p value of 0.05 or smaller and a log2 fold change ≤ − 0.5, were subjected to a GO term analysis using clusterProfiler (version 4.4.4) for R [32]. Adjusted p values of 0.05 or smaller were considered statistically significant; graphs were created using ggplot2 for R [33].

### Alternative splicing

Alternatively spliced exons were detected from RNA-Seq data using rMATS Turbo 4.1.2 for python 3.9 with the settings: read length 105, paired end reads, allow detection of novel splice sites, forbid clipping, and allow variable read lengths [34]. Results were filtered for relevant splicing events by coverage (at least 20 total junction reads per event), statistics (false discovery rate of 0.05 or smaller was considered statistically significant), and by the difference in the inclusion level (a difference of 0.1, which equates to 10%, was considered relevant) using dplyr (version 1.1.2) for R, and results were visualized using dot plots from ggplot2 (R) or Sashimi plots created with rmats2sashimiplot (version 2.0.4; python 2.7) [33, 35]. Afterward, alternatively spliced genes were subjected to a GO term analysis as described above. The lists of alternatively spliced genes were also scanned for WNT-associated genes using the PANTHER database (Version 17.0) through the web interface [36, 37].

### Endpoint PCR

Alternative splicing of CLK1 was additionally investigated using endpoint PCR to verify sequencing results. For this, HUVECs were treated with 10-µM C81, MU1210, or vehicle control for 6 h, after which RNA was isolated and reverse transcribed to cDNA as described above. Subsequently, cDNA was subjected to endpoint PCR using GoTaq DNA Polymerase (Promega, Wisconsin, USA) according to the manufacturer’s instructions with 35 cycles of the following program: initial denature: 95 °C, 2 min; denature 95 °C 1 min; annealing 56 °C 1 min; extension 72 °C 30 s; and final extension 72 °C 5 min. The length of the amplicons was then determined using 3% agarose gels (Thermo Scientific) supplemented with ethidium bromide (Carl Roth) and using a 100-bp ladder (NEB) as a reference. After electrophoresis in TBE buffer, DNA was visualized in the gel with an Azure C200 (Azure Biosystems, Dublin, California, USA) gel imaging system. Primers can be found in supplementary Table 1; an amplicon length of 177 bp indicated exon skipping and a length of 268 bp indicated exon inclusion.

### Cloning

The TCF/LEF and delTCF/LEF reporter gene plasmids were cloned into a sleeping beauty transposon backbone [38], kindly provided by Prof. Rolf Marschalek (Institute of Pharmaceutical Biology, Goethe University Frankfurt, Frankfurt, Germany), using the promoter, TCF/LEF response elements and luciferase sequence of the pGL4.49 Plasmid (Promega). For delTCF/LEF plasmids, the response elements were deleted from the sequence. The backbone contains an eGFP sequence behind a constitutive promoter, which serves as a transfection and selection control. The promoter region and luciferase sequence of pGL4.49, in addition to the sleeping beauty backbone, were linearized using PCR with the primers found in supplementary Table 1, with an overlap between backbone and inserts. This was done using the Q5 High-Fidelity DNA Polymerase (NEB) using the following program: initial denature: 98 °C, 30 s; denature 98 °C 10 s; annealing 64 °C 30 s; extension 72 °C 1 min for inserts, 3 min 40 s for the backbone; and final extension 72 °C 2 min. The linearized fragments were purified by agarose gel electrophoresis followed by gel extraction (Zymoclean Gel Extraction Kit, Zymo Research, Freiburg, Germany) and subsequently assembled using the NEBuilder Kit (NEB) according to the manufacturer’s instructions. The assembled plasmids were transformed into chemically competent DH10*β* *E. coli* cells and cultivated at 37 °C on agarose overnight. On the following day, clones were picked and cultivated overnight in a 2-ml liquid culture at 37 °C, 180 rpm. Plasmids were isolated using the GeneJET Miniprep Kit (Thermo) and were sequenced using Sanger sequencing at Microsynth Seqlab (Tübingen, Germany). Correct clones were grown in 100-ml cultures, and plasmids were isolated using the PureYield Plasmid Midiprep System (Promega). Subsequently, remaining endotoxins were removed using the MiraCLEAN Endotoxin Removal Kit (Mirus Bio, Madison, Wisconsin, USA) according to the manufacturer’s instructions.

### Luciferase reporter gene assay

HMEC-1 (1 million cells) were transfected with either TCF/LEF or delTCF/LEF plasmids, together with a SB100x transposase plasmid kindly provided by Dr. Eric Kowarz (Institute of Pharmaceutical Biology, Goethe University Frankfurt, Germany), at a ratio of 19:1, using the Nucleofector IIb device (Lonza, Basel, Switzerland) and the AMAXA HUVEC Nucleofector Kit and program A-034 according to the manufacturer’s instructions. For each transfection, 5 µg of total DNA were used. Transfected cells were transferred to a collagen G (Sigma-Aldrich)-coated well of a 6-well plate, cultivated for 2 days in fully supplemented ECGM (PELOBiotech), and subsequently selected using puromycin at 1–3 µg/ml for up to 2 weeks. Transfection and selection efficiencies were routinely checked using a Leica DMI6000 B Fluorescence Microscope (Leica Microsystems, Wetzlar, Germany). After successful selection, cells were seeded onto 48-well plates (Greiner Bio-One) and grown to confluency. Confluent cells were serum starved overnight (1% FCS in M199), then pretreated with the indicated concentrations of C81 for 30 min, and after which *β*-catenin signaling was induced using the GSK3 inhibitor LY2090314 at 30 nM. After 6 h, the incubation was stopped, and cells were lysed using 65-µl passive lysis buffer (Promega). 10 µl of the lysis solution was transferred to a white 96-well plate (Thermo Scientific). Luminescence was induced using the firefly substrate of the Luciferase Assay System (Promega) and measured using a plate reader (Tecan).

### Statistics

Graphs and statistics of all experiments, except RNA-Seq experiments, were created using GraphPad Prism 10.0.2 (Dotmatics, Boston, Massachusetts, USA). Generally, experiments were carried out in 3 or more independent replicates, and statistical significance was calculated using an unpaired 2-tailed students *t* test (experiments containing 2 groups) or an unpaired 1-way or a 2-way ANOVA (experiments with 3 or more groups). For ANOVA, post hoc analysis was performed to detect significant differences between specific datasets. Unless specified otherwise, this was done using Dunnett’s post hoc test, comparing treatments and negative controls to the single positive control. To improve readability, statistically significant differences, as defined by a p value of 0.05 or smaller, were marked with a single asterisk or other specified symbol, regardless of significance level, whereas non-significant differences are not marked at all.

## Results

### C81 inhibits angiogenesis in vivo

**Fig. 1:**
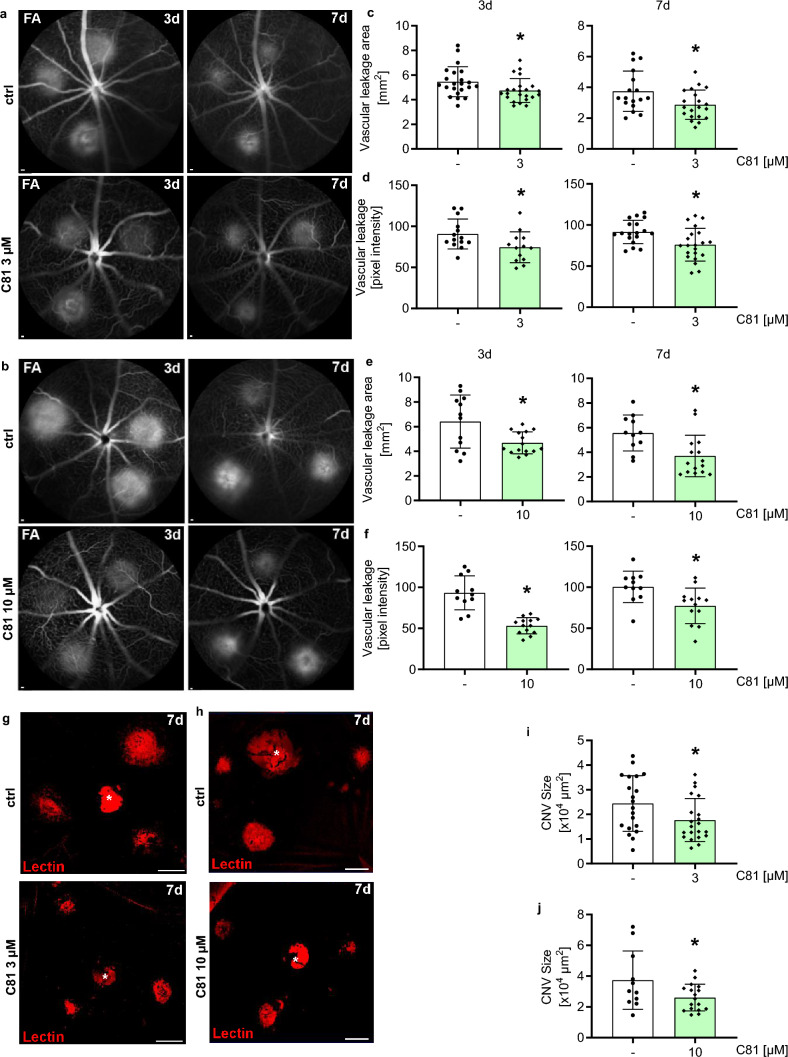
C81 limits laser-induced vascular leakage and pathological CNV in mice. **a**, **b** Representative late-phase fundus fluorescein angiography (FA) images at indicated time points post-laser injury. Scale bar: 200 μm. **c**–**f** Quantification of vascular leakage area (**c**, **e**) and vascular leakage intensity (**d**, **f**) after laser-induced CNV. **g**/**h** Representative laser-induced CNV stained with isolectin B4 in RPE/choroidal flat mounts. Scale bar: 100 μm. **i**/**j** Quantification of laser-induced CNV area in RPE/choroidal flat mounts. **c**–**f**/**i**, **j** Data are shown as mean ± SD; unpaired two-tailed students *t* test, ∗*p*  ≤ 0.05, **c**, **d**, **i** *n*  = 13–23 eyes, **e**, **f**, **j** *n* = 11–17 eyes

To test whether C81 inhibits angiogenesis, we applied it intravitreally in mice in a laser choroidal neovascularization (CNV) model, an established system to study key aspects of wAMD [1]. Bruch’s membrane was ruptured by a 532-nm laser, causing photocoagulation and subsequent angiogenesis. As can be seen in Fig. 1, both 3 and 10 µM of C81 significantly reduced vessel leakage as measured by fundus fluorescein angiography (FFA) in both area (Fig. 1c, e) and intensity (Fig. 1d, f) and 3 and 7 days after the laser injury. Especially for 10 µM of C81, the effects on vascular leakage intensity seemed to be more pronounced after 3 days compared to 7 days, which could be explained by elimination of the compound from the eye, as C81 is a small molecule [39]. Additionally, the fact that 10 µM of C81 had stronger effects than 3 µM indicated that C81’s effects were concentration dependent. Because vascular leakage is a marker for angiogenic activity of endothelial cells in CNV [40], this was a first indication of anti-angiogenic properties of C81. These results were confirmed by isolectin B4 staining of the RPE/choroidal flat mounts, which revealed that C81 reduced endothelial infiltration after laser-induced photocoagulation (Fig. 1i, j), further manifesting that the compound inhibited retinal angiogenesis in response to photocoagulation. There was initially no detectable difference in laser spot size between C81 treatment and control (Supp. Fig. 2a–f), but the spot size seemed to decrease slightly faster in eyes treated with 10 µM of C81 as compared to the vehicle control-treated eyes (Supp. Fig. 2g, h). Overall, these results indicate that C81 inhibits angiogenesis in vivo.

### C81 inhibits angiogenic processes ex vivo and in vitro

**Fig. 2:**
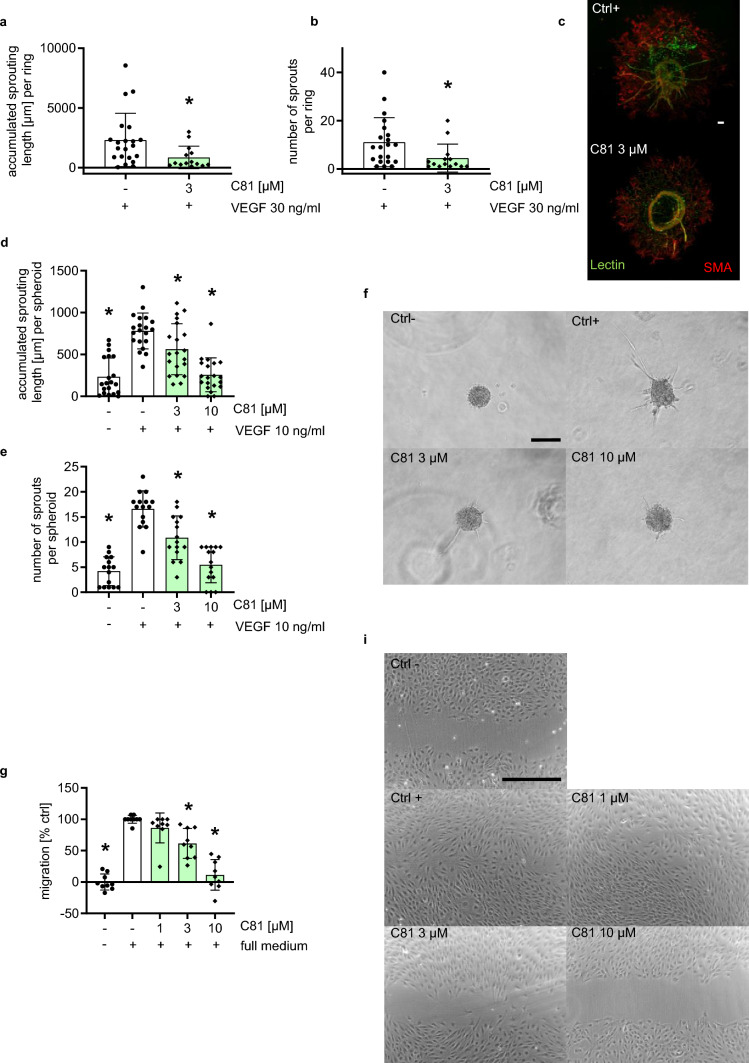
C81 inhibits angiogenic key steps ex vivo and in vitro.  **a**, **b** Quantification of VEGF stimulated mouse aortic rings treated for 3 days with 3-µM C81 for **a** accumulated length of sprouts per ring and **b** number of sprouts per ring. **c** Representative images of aortic rings stained with isolectin 1 (green) and anti-α-SMA (red) after treatment. **d**, **e** Quantification of HUVEC spheroids pretreated for 30 min with the indicated concentrations of C81/vehicle control and stimulated with VEGF for 20 h for accumulated sprouting length per spheroid (**d**) and number of sprouts per spheroid (**e**). **f** Representative images of collagen-embedded HUVEC spheroids at the end of treatment. **g** Quantification of scratches for closed surface area under treatment of C81, negative ctrl served as a baseline. **h** Representative images of scratches after incubation. **a**, **b**, **d**, **e**, **g** Data are represented as mean ± SD, *n* = 3 mice (**a**, **b**) or donors (**d**, **e**, **f**). **a**, **b** Unpaired, two-tailed students *t* test, **d**, **e**, **f** one-way ANOVA with Dunnett’s post hoc test, ∗*p*  ≤ 0.05 compared to VEGF ctrl (**a**, **b**, **d**, **e**) or vehicle ctrl (**g**). **c**, **f**, **h** Scale bar represents 100 μm

Because VEGF is a crucial mediator of angiogenesis in CNV [40], we subsequently tested whether C81 affects VEGF-driven neovessel formation in an ex vivo mouse aortic ring model. This model allows for specific, isolated investigation of pro-angiogenic growth factors, while still being a close mimic of physiological angiogenesis [1]. Figure 2a–c shows that C81 significantly reduced the capability of aortic rings to form new sprouts, as both total sprout length per ring (Fig. 2a) as well as the number of sprouts per ring (Fig. 2b) was significantly downregulated when VEGF-activated rings were treated with 3 µM of C81. This, in combination with the results from the laser CNV model, led us to believe that C81 is an inhibitor of VEGF-driven angiogenesis. In order to investigate which cellular processes are affected by C81 and by which mechanisms, we applied various in vitro models for different steps of the angiogenic cascade. Generally, angiogenesis occurs through several distinct cellular processes [41], of which we analyzed the impact of C81: sprout and tube formation, chemotactic and chemokinetic migration, and proliferation. Most importantly, C81 drastically reduced the capability of HUVECs to form sprouts from 3-dimensional spheroids when stimulated with VEGF (Fig. 2d–f), both in number of sprouts per spheroid (Fig. 2e) as well as accumulated sprouting length per spheroid (Fig. 2d). At 10 µM of C81, sprouting was blocked down to control levels. Subsequently, we found that HUVECs migrated less in a scratch model testing for chemokinetic migration (Fig. 2g, i) when subjected to C81, especially at the higher concentration of 10 µM. Additionally, HUVEC proliferation was inhibited by C81 with an IC_50_ of 3.73 µM (Supp. Fig. 3a). This was associated with an arrest in the G0/G1 stage of the cell cycle at lower concentrations of C81 as well as an additional arrest in the G2 phase at 10 µM (Supp. Fig. 3b). To test for HUVEC migration toward a chemoattractant, FCS was chosen and applied in a Boyden chamber setup (Supp. Fig. 3c) and a live-cell chemotaxis assay (Supp. Fig. 3d). While C81 inhibited directed migration in both assay setups, the live-cell chemotaxis setup revealed that C81 mainly influenced the capability of HUVECs to migrate toward a higher FCS concentration, as indicated by the forward migration index toward the chemoattractant (FMI:II). However, while directedness and Euclidean distance seemed to be weakly affected, albeit not statistically significant, no effect was observable on the accumulated distance and velocity (Supp. Fig. 3d) of migrating HUVECs. Additionally, we also investigated the effect that C81 had on tube formation in HUVECs. At 1, 3, and 10 µM, C81 strongly and significantly hindered HUVECs in forming capillary like structures on Matrigel (Supp. Fig. 3e–g). Moreover, we also performed selected key assays with the microvascular cell line HMEC-1 [28], as physiological angiogenesis is mediated by the microvascular endothelium (Supp. Fig. 4) [1] to confirm the results obtained in HUVECs. In accordance with HUVEC data, C81 impeded VEGF-triggered sprouting from HMEC-1 spheroids (Supp. Fig. 4a–c), reduced chemokinetic migration (Supp. Fig. 4d, e), and inhibited HMEC-1 proliferation with an IC_50_ of 4.53 µM (Supp. Fig. 4f). Overall, C81 was found to inhibit angiogenesis through a combined inhibition of migration, proliferation, tube formation, and sprouting of endothelial cells.

### C81 impedes endothelial VEGF/VEGFR2 signaling by inhibiting VEGFR2 protein and mRNA expression

**Fig. 3:**
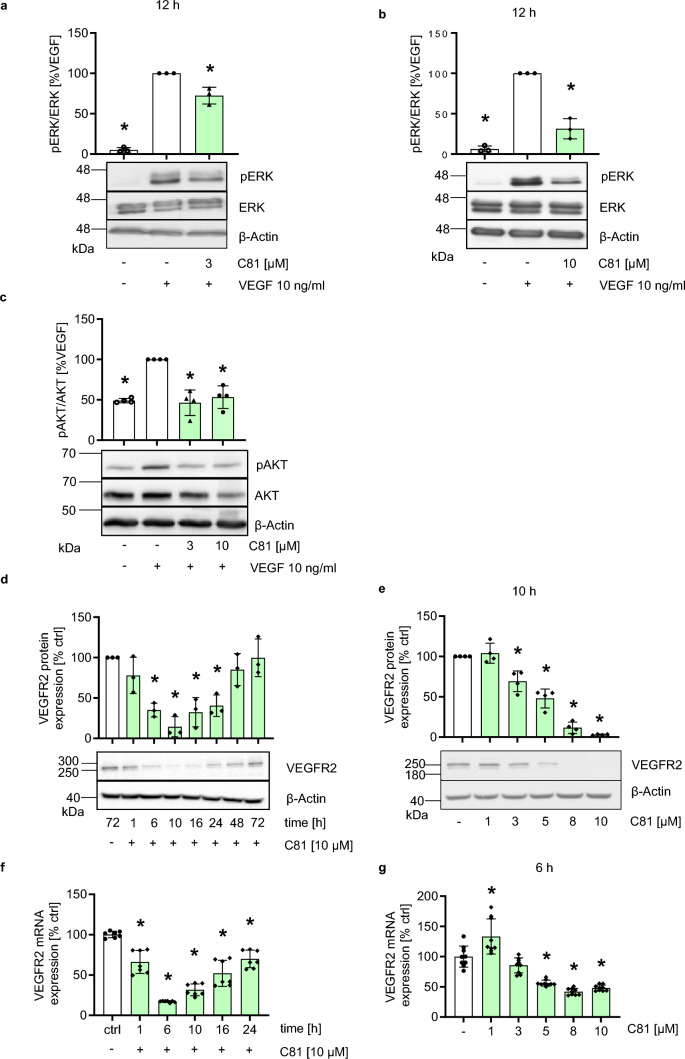
C81 inhibits VEGF signaling by downregulating VEGFR2 protein and mRNA expression. **a**, **b** Semiquantitative evaluation of ERK activation by 5-min VEGF stimulation under treatment of 12 h 3-µM (**a**) or 10-µM (**b**) C81 in serum starvation medium using western blot. **c** Semiquantitative evaluation of AKT activation by 15-min VEGF stimulation under treatment of 10 h 3 and 10 µM in serum starvation medium using western blot. **a**, **b**, **c** Cells were serum starved overnight before treatment with C81. **d**, **e** Semiquantitative assessment of VEGFR2 protein expression at 10-µM C81 for 1–72 h (**d**) or at 10 h with 1–10-µM C81 (**e**) using Western blot. **f**, **g** Relative quantification of VEGFR2 mRNA expression at 10 µM for 1–24 h or at 6 h with 1–10-µM C81. **a**–**e** Data are measured by densitometry using Fiji/ImageJ, normalized to ERK and *β*-actin (**a**, **b**), AKT and *β*-actin (**c**), *β*-actin (**d**, **e**),  or GAPDH (**f**, **g**) and represented as mean ± SD, **a**, **b**, **d**, **f**, **g** *n* = 3 donors, **c**, **e** *n* = 4 donors, **a**–**g** one-way ANOVA with Dunnett’s post hoc test, ∗*p*  ≤ 0.05 vs. VEGF ctrl (**a**–**c**) or vehicle ctrl (**d**–**g**)

As we focused on VEGF-driven angiogenesis in the applied functional assays, we decided to look further into the VEGF/VEGFR2 signaling cascade in endothelial cells, which is a crucial axis in VEGF-dependent angiogenesis [42], in order to get insights into the mechanism of action of C81. When activated by VEGF, the receptor tyrosine kinase (RTK) VEGFR2 dimerizes, auto-phosphorylates, and thus activates multiple downstream signaling pathways, e.g., mitogen-activated protein kinase (MAPK) signaling, in which the extracellular signal-regulated kinase 1 and 2 (ERK1/2) are activated by phosphorylation and protein kinase B (PKB/AKT) signaling, which then initiates pro-angiogenic cell functions by activating transcription factors [43–45]. As a surrogate parameter for VEGFR2 activation, we chose to investigate VEGF-induced ERK phosphorylation and VEGF-induced AKT phosphorylation. C81 significantly inhibited VEGF-induced ERK (Fig. 3a, b) and AKT (Fig. 3c) activation at both 3 µM and 10 µM. Subsequently, we analyzed the VEGFR2 protein expression and observed that 10 µM of C81 strongly reduced VEGFR2 protein (Fig. 3d) and mRNA expression (Fig. 3f) in a time-dependent manner. Effects were particularly strong at 10 h of treatment for VEGFR2 protein expression and at 6 h for VEGFR2 mRNA expression. Additionally, we chose these timepoints to test C81 concentrations between 1 and 10 µM and found that C81 downregulated VEGFR2 protein (Fig. 3e) and mRNA expression (Fig. 3g) in a concentration-dependent manner.

### CLK inhibitors impede VEGFR2 protein expression, whereas DYRK2 or PIM3 inhibition does not

**Fig. 4:**
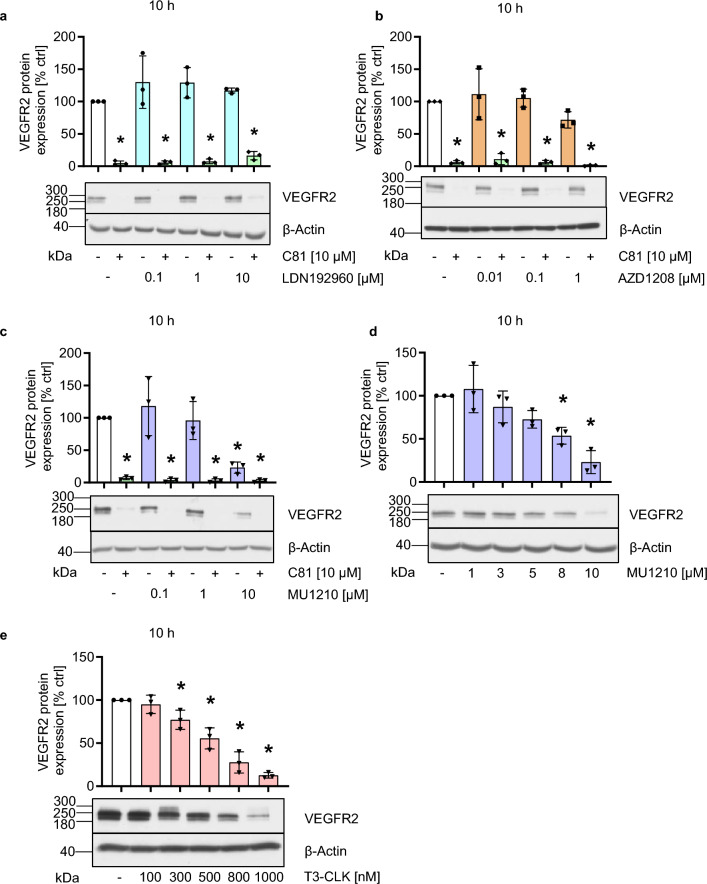
CLK inhibitors impede VEGFR2 protein expression, while DYRK2 and PIM inhibitors do not. **a**–**e** Semiquantitative western blot analysis of VEGFR2 protein expression under treatment of 10 h of DYRK2 **a,** PIM **b,** or CLK **c**–**e** inhibitors in increasing concentrations in combination with C81 (**a**–**c**) or without C81 (**d**, **e**). **a**–**f** Data are measured by densitometry using Fiji/ImageJ, normalized to *β*-actin and represented as mean ± SD, *n* = 3 donors, one-way ANOVA with Dunnett’s post hoc test, ∗*p*  ≤ 0.05 vs. vehicle control

We previously established C81 as an inhibitor of the cdc2-like kinases (CLKs) 1 and 4, the dual-specificity tyrosine-regulated kinase 2 (DYRK2), and Pim-3 proto-oncogene (PIM3) [13]. As the role of these kinases in the expression of VEGFR2 has, to the knowledge of the authors, not been investigated so far, we chose to apply established and selective pharmacological inhibitors to HUVECs to test whether they impede the protein expression of VEGFR2. As an inhibitor of DYRK2, LDN192960 was used in concentrations up to 10 µM, which did not alter VEGFR2 protein expression after 10 h of treatment (Fig. 4a) [46]. Similarly, the PIM1-3 inhibitor AZD1208 had no effect on VEGFR2 protein expression after 10 h of treatment in concentrations up to 1 µM (Fig. 4b) [47]. In contrast to LDN192960 and AZD1208, the CLK1, 2, and 4 inhibitor MU1210 was able to achieve a similar downregulation of VEGFR2 protein expression (Fig. 4c/d) with a similar concentration dependency as C81 (Fig. 4d) [48]. To further confirm these results, we applied the pan-CLK inhibitor T3-CLK, which likewise showed a similar concentration-dependent downregulation of VEGFR2 protein expression, but proved to be more potent than C81 and MU1210 (Fig. 4e) [49]. These results indicate that CLK inhibition might be the main cause of C81’s effects on VEGFR2 expression.

### MU1210 and T3-CLK inhibit angiogenesis-related cellular functions in vitro

**Fig. 5:**
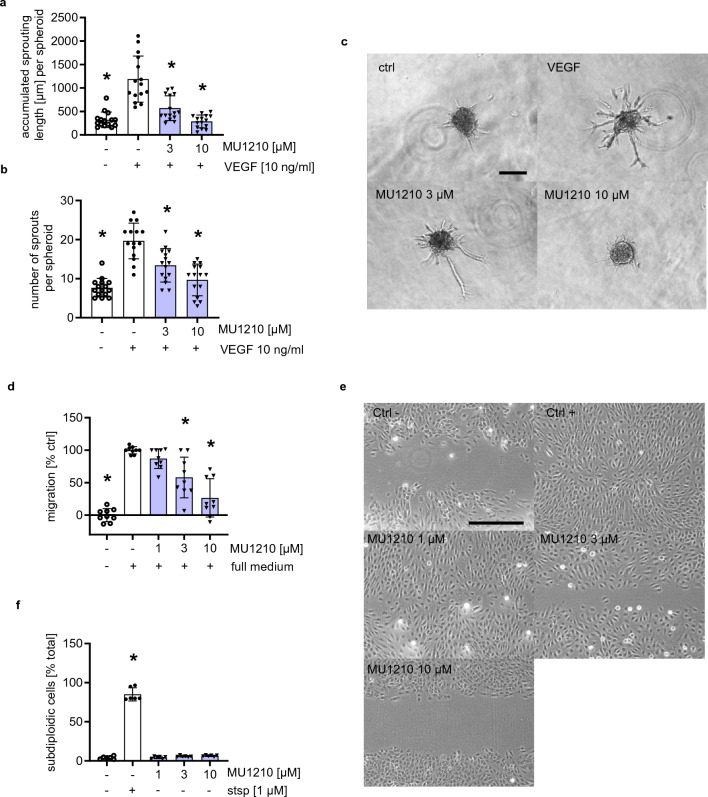
MU1210 inhibits angiogenic key steps in vitro without inducing apoptosis. **a**, **b** Quantification of HUVEC spheroids pretreated for 30 min with the indicated concentrations of MU1210/vehicle control and stimulated with VEGF for 20 h for accumulated sprouting length per spheroid (**a**) and number of sprouts per spheroid (**b**). **c** Representative images of collagen-embedded HUVEC spheroids at the end of treatment. **d** Quantification of scratches for closed surface area under treatment of MU1210, negative ctrl served as a baseline. **e** Representative images of scratches after incubation. **f** Quantification of apoptosis under 24-h treatment of indicated concentrations of MU1210. **a**, **b**, **d**, **f** Data are represented as mean ± SD, one-way ANOVA with Dunnett’s post hoc test, *n* =3, ∗*p* ≤ 0.05 compared to VEGF ctrl (**a**, **b**) or vehicle ctrl (**d**, **f**). **c**, **e** Scale bar represents  100 μm

**Fig. 6:**
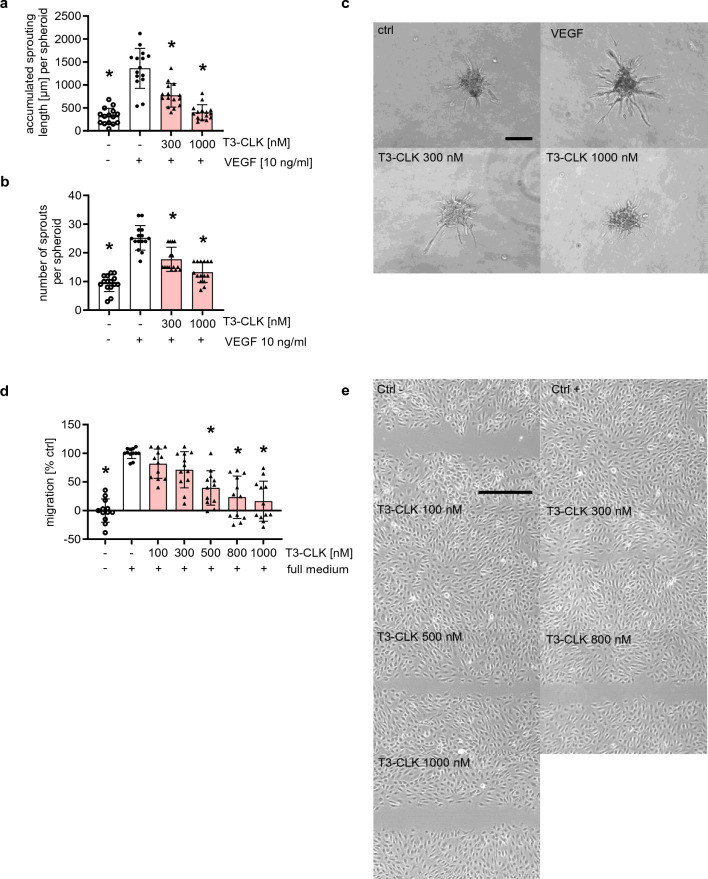
T3-CLK inhibits angiogenic key steps in vitro. **a**, **b** Quantification of HUVEC spheroids pretreated for 30 min with the indicated concentrations of T3-CLK/vehicle control and stimulated with VEGF for 20 h for accumulated sprouting length per spheroid (**a**) and number of sprouts per spheroid (**b**). **c** Representative images of collagen-embedded HUVEC spheroids at the end of treatment. **d** Quantification of scratches for closed surface area under treatment of T3-CLK, negative ctrl served as a baseline. **e** Representative images of scratches after incubation. **a**, **b**, **d** Data are represented as mean ± SD, one-way ANOVA with Dunnett’s post hoc test, ∗*p*  ≤ 0.05 compared to VEGF ctrl (**a**, **b**) or vehicle ctrl (**d**). **a**, **b** *n* = 3, **d** *n*  = 4 donors. **c**, **e** Scale bar represents 100 μm

To confirm that MU1210 and T3-CLK not only phenocopy C81’s effects on VEGFR2 expression, but also the effects on functional models of angiogenesis, we tested these inhibitors in the scratch assay and the spheroid sprouting assay. Both compounds inhibited VEGF-induced sprouting from HUVEC spheroids in the applied concentrations of 3 and 10 µM of MU1210 (Fig. 5a–c) and 300 and 1000 nM of T3-CLK (Fig. 6a–c). In the concentrations of 3 and 10 µM, MU1210 inhibited the undirected migration in HUVECs (Fig. 5d, e), as did T3-CLK in concentrations of 500, 800, and 1000 nM (Fig. 6d, e). Additionally, MU1210 did not induce apoptosis in HUVECs in concentrations of up to 10 µM over an incubation period of 24 h (Fig. 5f), indicating comparably low cytotoxicity to C81 [13]. In summary, these results show that CLK inhibitors inhibit angiogenesis-related cellular processes in vitro*.*

### C81 is a pan-CLK inhibitor and C81 and MU1210 exert similar effects on p-SR proteins

**Fig. 7:**
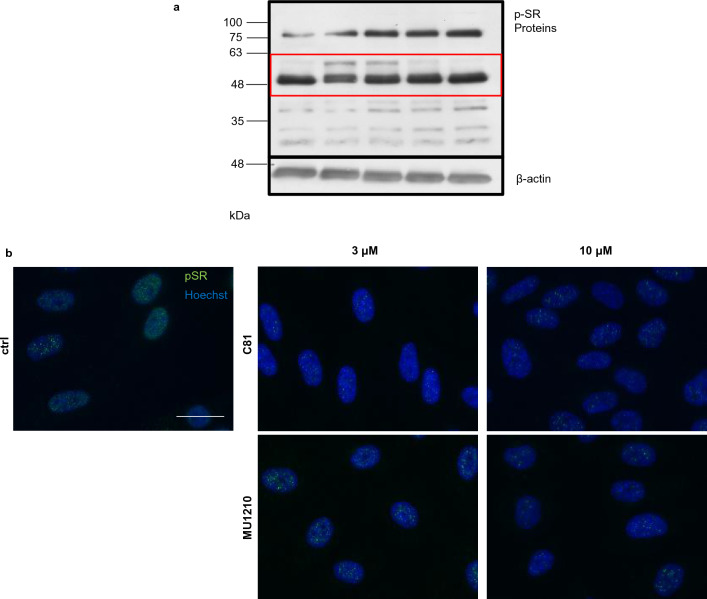
CLK inhibition by C81 and MU1210 exerts similar effects on phosphorylated serine- and arginine-rich splicing factors (p-SR proteins). **a** Western blot analysis of p-SR proteins. In the red-marked region, an electromobility shift of SRSF6 can be observed when treated with 10-µM C81 or MU1210 for 6 h. **b** Immunofluorescence pictures of HUVECs stained with Hoechst 33342 for the nuclei and anti-p-SR proteins to mark interchromatin granule clusters. Altered distribution can be observed when HUVECs are treated with C81 or MU1210 for 6 h. **a**, **b** One representative donor out of 3 independent experiments is shown, **b** scale bar represents 25 μm

As reported above, T3-CLK had a stronger potency, while C81 and MU1210 showed effects on VEGFR2 expression and HUVEC sprouting at similar concentrations. For this reason and due to the fact that MU1210 has been characterized more thoroughly compared to T3-CLK in regards to potential off-targets [15], we focused on MU1210 as the reference compound. However, our previously published data on CLK inhibition exerted by C81 is limited to CLK1 and CLK4 [13], whereas it is known that MU1210 inhibits CLK1, CLK2, and CLK4 [48] and T3-CLK inhibits all isoforms, but with a higher IC_50_ for CLK3 compared to the others [15, 49]. To make up for this lack of data, we established K_D_ values for all CLKs for C81 using the Eurofins DiscoveRx KdELECT platform. Measurements are listed in Table 2 and revealed C81 to be a pan-CLK inhibitor. Because K_D_ values were determined for C81, while IC_50_ values are published for MU1210 [48], we set out to investigate how comparable both inhibitors are affecting direct downstream targets of CLKs in HUVECs at 3 and 10 µM. To do this, we decided to asses effects on phosphorylated serine- and arginine-rich splicing factors (p-SR proteins), which are well-studied downstream targets of CLKs and have been used as evidence for CLK inhibition in multiple publications [14, 15, 18, 49–51]. Both C81 and MU1210 induced a comparable electromobility shift in what has previously been described to be SRSF6 [14, 18, 49–51] (Fig. 7a), similar to other previously published CLK inhibitors [49, 50]. In both cases, the shift is barely visible at 3 µM and very pronounced at 10 µM. Accordingly, C81 and MU1210 also altered the distribution of p-SR proteins as interchromatine granule clusters (IGCs) in nuclei similar to previously published CLK inhibitors (Fig. 7b, Supp. Fig. 5) [14, 18, 50]. This shows that C81 and MU1210 had very similar effects at similar concentrations on downstream targets of CLKs, which is in accordance with the similar concentration dependencies observed in the VEGFR2 expression and in vitro models of angiogenesis.

**Table 2:** *K*_D_ values for C81 against all 4 CLK isoforms

Kinase	*K*_D_ [nM]
CLK1	520
CLK2	960
CLK3	2300
CLK4	1900

### CLKs are crucial for VEGFR2 expression and HUVEC spheroid sprouting

**Fig. 8:**
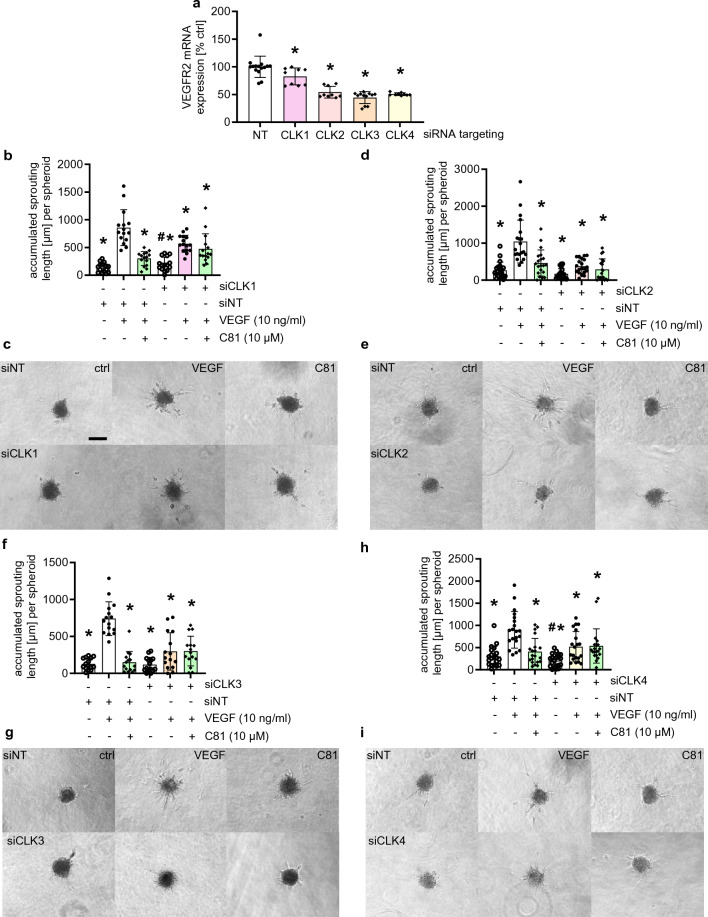
Knockdown of CLKs, especially 2–4, inhibits VEGFR2 mRNA expression and VEGF-induced spheroid sprouting. **a** Relative quantification of VEGFR2 mRNA expression, normalized to GAPDH, 72 h after the indicated knockdown was induced in HUVECs. **b**, **d**, **f**, **h** Quantification of HUVEC spheroids embedded into collagen 72 h after the indicated knockdown was induced, pretreated for 30 min with the indicated concentrations of C81/vehicle control and stimulated with VEGF for 20 h for accumulated sprouting length per spheroid. **c**, **e**, **g**, **i** Representative images of collagen-embedded HUVEC spheroids at the end of treatment. **a**, **b**, **d**, **f**, **h** Data are represented as mean ± SD, *n* = 3 donors (**a**, **b**, **f**) or *n* = 4 donors (**d**, **h**) one-way ANOVA with Dunnett’s post hoc test (**a**) or Tukey’s post hoc test (**b**, **d**, **f**, **h**), ∗*p*  ≤ 0.05 compared to non-targeting control (**a**) or VEGF non-targeting control (**b**, **d**, **f**, **h**); **b**, **d**, **f**, **h** #*p*  ≤ 0.05 compared to knockdown cells stimulated with VEGF, only used to compare treatments of knockdown cells. **c**, **e**, **g**, **i** Scale bar represents 100 μm

While T3-CLK and MU1210 are among the most selective currently available CLK inhibitors, they still have multiple off-targets [15, 48, 49]. Additionally, the investigation of the role of individual CLKs is very difficult when using chemical probes, because they usually target more than one CLK [15]. We therefore investigated the degree of regulation of VEGFR2 expression and spheroid sprouting in HUVECs by performing knockdown experiments using siRNA against each CLK isoform. The knockdown of all CLKs was effective with less than 50% remaining mRNA expression (Supp. Fig. 6a–d); however, CLK3 knockdown seemed to be more effective than the knockdown of all other CLKs (Supp. Figure 6c). Nevertheless, 72 h after transfection VEGFR2 mRNA expression was downregulated in all CLK knockdowns. However, while statistically significant, the knockdown of CLK1 only evoked a weak inhibition of VEGFR2 mRNA expression (Fig. 8a) to about 80%. In contrast, CLK2-4 knockdowns all showed about 50% or less remaining VEGFR2 mRNA expression compared to the non-targeting control (Fig. 8a). This lower expression of VEGFR2 also affected the spheroid sprouting assay (Fig. 8b–I, Supp. Fig. 6e–h). Here, all CLK knockdowns strongly inhibited spheroid sprouting, with CLK1 knockdown again showing the weakest effects (Fig. 8b, c, Supp. Fig. 6e), while CLK2-4 knockdowns showed a very strong reduction of sprouting (Fig. 8d–I, Supp. Fig. 6f–h). Additionally, none of the knockdowns indicated an increased effect in combination with C81 (Fig. 8b–I, Supp. Fig. 6e–h), which further proved that the respective kinases are indeed the responsible targets for the effects exerted by C81. In summary, CLK2-4 especially seem to be important kinases in the regulation of VEGFR2 expression and angiogenesis-related cellular processes. They are very likely to be the primary targets of C81 and other CLK inhibitors in the inhibition of angiogenesis.

### CLK inhibitors do not significantly alter the splicing of VEGFR2

**Fig. 9:**
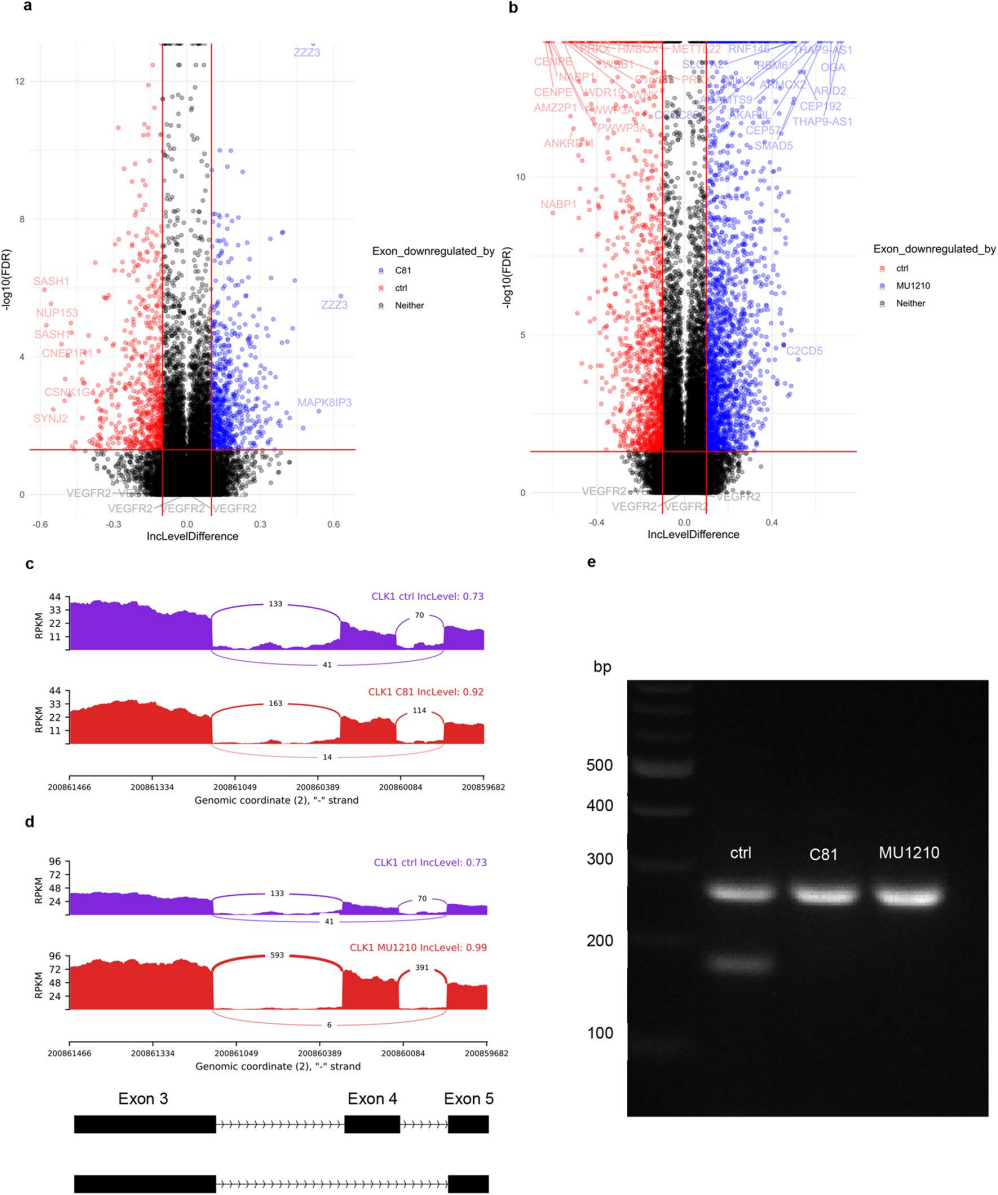
C81 and MU1210 alter splicing in HUVECs, but not of VEGFR2. **a**, **b** Results of isoform analysis using rMATS turbo of all alternative splicing events in HUVECs treated with 10-µM C81 (**a**) or 10-µM MU1210 (**b**) for 6 h, and red lines separate significant from non-significant events. Events with an inclusion level difference of 0.5 or higher as well as all events related to VEGFR2 are labeled. **c**, **d** Sashimi plots of increased exon 4 retention in CLK1 mRNA, which is typical of CLK inhibitors, for C81 (**c**) and MU1210 (**d**). **e** Verification of increased exon 4 retention detected in the RNA-seq data through endpoint PCR and agarose gel electrophoresis of cDNA of HUVECs treated for 6 h with 10-µM C81, 10-µM MU1210, or vehicle control. The larger amplicon (268 bp) includes exon 4, whereas the smaller amplicon (177 bp) does not. **a–d** Graphs depict the averages of *n* = 3 donors, **e** one representative picture out of 3 independent experiments is shown

Because the main known function of CLKs is the phosphorylation of splicing factors, which is important for the splicing process, CLK inhibitors are often shown to exert their effects through alterations of splicing [14, 15, 18, 49–51]. Therefore, we hypothesized that the CLK inhibitors tested in this study might alter the splicing of VEGFR2 pre-mRNA, which then could lead to reduced VEGFR2 protein expression through a variety of mechanisms. However, while short read next-generation RNA-sequencing (RNA-Seq) revealed that both C81 and MU1210 alter the splicing of multiple mRNAs in HUVECs, all detected alternative splicing events related to VEGFR2 showed low differences to the control and were not statistically significant (Fig. 9a, b), indicating that alternative splicing of VEGFR2 is not a relevant mechanism of these compounds. To validate the sequencing results, we analyzed at the alternative splicing of exon 4 in CLK1 mRNA, which has been shown to be affected by CLK inhibitors [52]. Both C81 and MU1210 strongly and significantly increased the retention of exon 4 in CLK1 mRNA, which can be seen in the Sashimi plots (Fig. 9c, d). Additionally, we confirmed this by performing endpoint PCR with primers in exon 3 and 5 and subsequent gel electrophoresis (Fig. 9e). The shorter variant, which is the one amplified from CLK1 mRNA without exon 4, is barely detectable when HUVECs are treated with 10 µM of either C81 or MU1210. It follows that our RNA-Seq approach was successful in identifying alternatively spliced mRNAs in HUVECs treated with C81 or MU1210; however, no direct effect on VEGFR2 mRNA could be observed, indicating that VEGFR2 is not downregulated due to direct changes in splicing.

### *β*-Catenin inhibition is partially responsible for C81-triggered effects

**Fig. 10:**
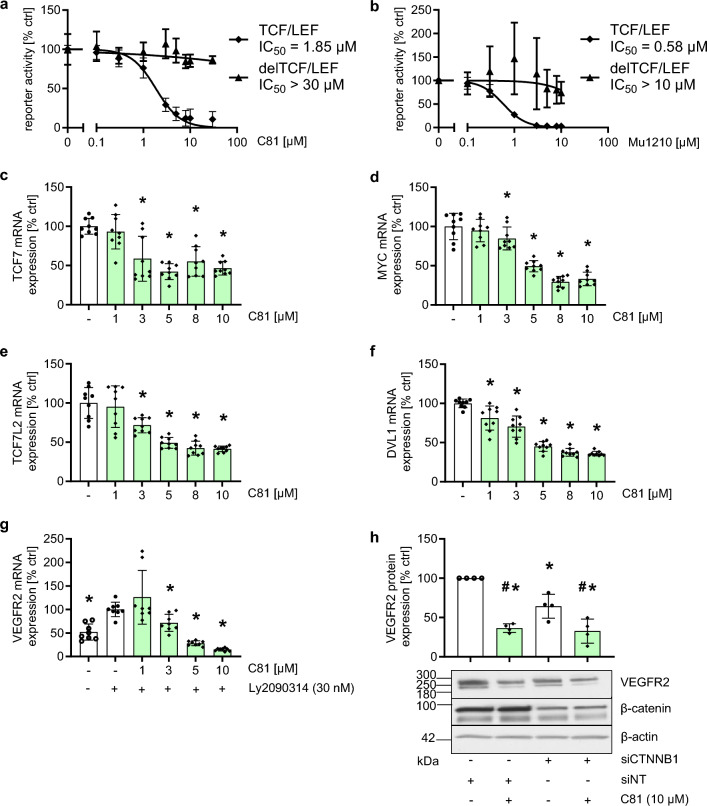
C81 and MU1210 inhibit *β*-catenin signaling and induction of *β*-catenin signaling induces, while knockdown of *β*-catenin reduces VEGFR2 expression. **a**, **b** Measurements of luciferase activity in a reporter gene assay with a TCF/LEF responsive promoter and a constitutive promoter using LY2090314 (30 nM) to induce *β*-catenin signaling and increasing concentrations of C81 (**a**) or MU1210 (**b**). **c**–**g** Relative expression of selected WNT/β-catenin related or WNT/*β*-catenin target genes (**c**–**f**) and VEGFR2 (**g**) in HUVECs treated with C81 in the indicated concentrations in full medium (**c**–**f**) or with indicated concentrations of C81 and LY2090314 in serum starvation medium (**g**) for 6 h was measured using qPCR with GAPDH as a reference. **h** Semiquantitative analysis of VEGFR2 protein expression, normalized to *β*-actin, using densitometry in Fiji/ImageJ in HUVECs 48 h after *β*-catenin knockdown was induced. C81 was added for the final 10 h of the incubation. **a**–**h** Data are depicted as mean ± SD, **a**, **b** IC_50_ values were calculated using a dose–response curve with a variable slope, **c**–**h** one-way ANOVA with Dunnett’s post hoc test (**c**–**g**) or Tukey’s post hoc test (**h**) ∗*p *≤ 0.05 compared to vehicle control (**c**–**f**),  LY2090314 (**g**),  or non-targeting control (**h**), **h** #p ≤ 0.05 compared to siCTNNB1. **a**–**g** *n* = 3, **h** *n* = 4

We continued to search for potential mechanisms of how C81 might affect VEGFR2 expression by performing a GO term analysis for biological processes related to genes downregulated by C81 treatment (Supp. Figure 7). Multiple processes were significantly affected, especially processes related to RNA splicing and post-translational modification of proteins. However, very few signaling pathways that could potentially affect VEGFR2 expression directly were significantly overrepresented in these genes. Most prominently, the WNT signaling pathway appeared multiple times in the list of affected biological processes. Interestingly, previous studies have shown that the WNT/glycogen synthase kinase-3*β*(GSK3*β*)/*β*-catenin axis can affect the expression of VEGFR2 in HUVECs and central nervous system endothelial cells, including retinal endothelial cells [20, 21]. Additionally, some CLK inhibitors have been characterized as WNT/GSK3*β*/*β*-catenin inhibitors [14, 18, 51]. We therefore suspected that CLK inhibitors applied in this study might downregulate VEGFR2 expression by inhibiting *β*-catenin activity. To investigate this hypothesis, we initially confirmed that both C81 and MU1210 inhibit *β*-catenin transcriptional activity in a TCF/LEF-dependent reporter gene assay (Fig. 10a, b). As this assay was performed using a GSK3 inhibitor to induce *β*-catenin-dependent activation of the reporter gene and because it was performed in HMEC-1 instead of HUVECs, we further tested whether C81 reduces the activity of the WNT/GSK3*β*/*β*-catenin axis by quantifying key pathway and/or target genes in HUVECs without specific stimulation of the pathway. Figure 10c–f shows that C81 strongly reduced the mRNA expression of transcription factor 7 (TCF7, Fig. 10c), MYC proto-oncogene (MYC, Fig. 10d), TCF7-like 2 (TCF7L2, Fig. 10e), and disheveled segment polarity protein 1 (DVL1 Fig. 10f) in HUVECs. MYC is an important *β*-catenin target gene [53], TCF7 and TCF7L2 are key transcription factors interacting with *β*-catenin and TCF7 is, in addition, a target gene of *β*-catenin-induced transcription [53, 54], and DVL1 is a key protein that inhibits the *β*-catenin destruction complex [55]. Furthermore, we wanted to investigate if *β*-catenin affects the expression of VEGFR2, to test whether this inhibition of *β*-catenin is responsible for the effects derived from CLK inhibitor treatment. Indeed, inducing *β*-catenin transcriptional activity with a GSK3*β* inhibitor significantly increased VEGFR2 mRNA expression, which was reversed by treatment with C81 (Fig. 10g). These results confirm the observations by Skurk et al. [20]. More importantly, however, knockdown of *β*-catenin (CTNNB1) significantly reduced VEGFR2 protein expression compared to non-targeting control (Fig. 10h) in HUVECs. The knockdown efficiency varied between donors (Supp. Figure 8); however, this did not correspond to differing effects on VEGFR2 expression. Interestingly, *β*-catenin knockdown in combination with C81 did not increase the effect of C81 (Fig. 10h). These data confirm that inhibition of *β*-catenin activity, derived from CLK inhibition, is at least partially responsible for the anti-angiogenic effects of C81. However, the mechanism through which CLK inhibitors decrease the activity of the WNT/GSK3*β*/*β*-catenin axis remains unclear in our opinion. While previous publications generally assume that alternative splicing of WNT pathway and/or target genes is the mechanism of action [14, 18, 51], we could not observe a statistical enrichment of WNT-related genes in a GO term analysis of alternative splicing events induced by treatment with either C81 (Supp. Table 2) or MU1210 (Supp. Table 3). Therefore, we believe that if alternative splicing is the mechanism through which CLK inhibitors affect WNT signaling, there needs to be one or a few genes alternatively spliced in such a way that it inhibits the cascade. In accordance with these previous publications [14, 18, 51], we could observe that both C81 and MU1210 affect the splicing of multiple WNT-related genes (Supp. Table 4, genes alternatively spliced by C81 and MU1210 treatment are underlined) and suggest that these alternative splicing events should be further investigated to uncover the precise mechanism of CLK inhibitor-derived WNT/GSK3*β*/*β*-catenin inhibition.

## Discussion

Angiogenesis is a crucial physiological process, especially in growth and wound healing, as it is necessary for the supply of nutrients and oxygen to the tissue [1, 2]. However, dysregulated angiogenesis is part of multiple pathophysiological conditions, most notably cancer, wet age-related macular degeneration (wAMD), and chronic inflammatory diseases, like rheumatoid arthritis or psoriasis [8]. In many cases, hyperactivation of the endothelium results in a large number of leaky blood vessels, which can contribute to the progression of the underlying disease [5–7]. In cancer and wAMD, inhibitors of angiogenesis have been applied with some success [7, 9]. However, inhibitors of angiogenesis provide limited efficacy, especially in wAMD, partly because underlying inflammation-related processes can still drive the disease [10]. Therefore, compounds that affect both angiogenesis and inflammation could potentially improve the treatment of wAMD as well as other diseases that depend on both.

C81, originally called 2-desaza-annomontine, is a synthetically accessible derivative of the plant alkaloid annomontine [12]. It has been shown to inhibit human CLKs, which we also confirmed and expanded on here, as well as PIM3 and DYRK2 [13]. Additionally, C81 and other CLK inhibitors impede various inflammatory processes, e.g., by downregulating TNFR1 in the endothelium [13] or by WNT/*β*-catenin inhibition in immune cells [14, 17, 51]. In this study, we demonstrate that C81 and other CLK inhibitors reduce angiogenesis-related cell functions by downregulating VEGFR2 in endothelial cells, which could also be replicated by CLK knockdowns, revealing CLK inhibitors to be a promising class of compounds in the treatment of diseases that depend on angiogenesis and inflammation.

Importantly, we could show that C81 reduced the leakage of fluorescein and the infiltration of endothelial cells into the RPE/choroid after laser-induced CNV in vivo, which indicates impaired CNV. It has been comprehensively shown that CNV at least partially depends on VEGF [40]. We further uncovered that C81 inhibited VEGF-driven neovessel formation in an ex vivo aortic ring model. Additionally, we applied in vitro models testing for migration, proliferation, tube formation, and sprout formation of endothelial cells, to investigate through which processes C81 affects angiogenesis. C81 inhibited all angiogenesis-related cellular functions that we tested for, like VEGF-triggered sprouting from EC spheroids, but also migration and proliferation of ECs, in both HUVEC and HMEC-1. Moreover, C81 also strongly impeded tube formation in HUVECs. In assays testing for undirected migration, proliferation, and sprouting, 10-µM C81 reduced angiogenic activity to about negative control levels, whereas the effects in assays testing for directed migration were not as strong. Nonetheless, these results indicate that C81 inhibited VEGF-induced angiogenesis through downregulation of the pro-angiogenic activity of endothelial cells.

Furthermore, we provide evidence that C81 hindered VEGF-induced signaling in endothelial cells through a substantial downregulation of VEGFR2, the main activating receptor for VEGF in the vascular endothelium [4], at protein and mRNA levels. This effect was time and concentration dependent and provided an explanation for the observed anti-angiogenic properties of C81. However, it should be noted that while among the most important, VEGF/VEGFR2 is not the only pro-angiogenic signaling system [8, 9]. In fact, while a lot of our functional assays focused on VEGF, we still observed strong effects of CLK inhibitors even when FCS or fully supplemented ECGM was used as the stimulant, for example, in the scratch assay or the tube formation assay. This indicates that CLK inhibitors might also affect pathways other than the VEGF/VEGFR2 axis, which should be further investigated to elucidate the full anti-angiogenic potency of these compounds.

When establishing the potential targets responsible for effects derived from C81, we applied established inhibitors of these kinases targeted by C81 and observed that only inhibitors of CDC2-like kinases inhibited VEGFR2 protein expression, while DYRK2 and PIM3 inhibitors did not significantly affect this expression. This was the crucial finding in establishing the hypothesis that CLK inhibition was the main reason for the C81-derived effects on angiogenesis. Moreover, the CLK1, 2, and 4 inhibitor MU1210 was about as potent as C81 in inhibiting angiogenesis-related cellular processes in vitro and in downregulating VEGR2 protein expression. The pan-CLK inhibitor T3-CLK was even more potent, which could potentially be explained by its greater affinity toward CLKs as compared to C81 and MU1210 [13, 15, 48, 49]. The comparable concentration dependency between MU1210 and C81 correlated with the fact that effects on p-SR proteins also were highly similar at identical concentrations, which substantiated the hypothesis that CLKs are the primarily responsible target for C81-derived effects. Finally, this hypothesis was confirmed by CLK knockdowns, especially of CLK2, CLK3, and CLK4, which strongly impeded spheroid sprouting and VEGFR2 mRNA expression. Therefore, it appears that CLKs play an important role in angiogenic processes through the regulation of VEGFR2 expression. However, several crucial points need to be kept in mind when discussing these results: It is, for example, difficult to deduce from our data which CLK isoform is the most important in regulating angiogenesis and VEGFR2 expression, even though it appears that CLK1 is the least important. This is mainly because knockdown efficiencies were not comparable between CLK isoforms, especially CLK3 showed a much stronger knockdown efficiency compared to CLK1, CLK2, and CLK4. Additionally, none of the applied CLK inhibitors, including C81, are specific for a CLK isoform [48, 49]. Moreover, no CLK knockdown was as effective in inhibiting spheroid sprouting as applying C81 to non-targeting siRNA-transfected cells, which indicated that inhibition of multiple CLKs could exert synergistical effects and/or that the knockdown of CLKs was not as effective in inhibiting their kinase activity as C81.

Furthermore, while we do believe to provide conclusive evidence that the major responsible targets for the C81-derived inhibition of angiogenesis are CLKs, involvement of the mentioned off-targets of C81 cannot be fully ruled out. In fact, PIM3 has been shown to play a role in angiogenesis-related cellular processes, especially spreading and migration of endothelial cells [56]. However, since the pan-Pim kinase inhibitor AZD1208 was unable to phenocopy the C81-derived downregulation of VEGFR2 protein expression, and because PIM3 has not been reported as an off-target for either T3-CLK or MU1210 [48, 49], we believe that the C81-mediated inhibition of PIM3 is not of major importance for the observed cellular effects. To our knowledge, no studies have ever reported impaired angiogenesis resulting from DYRK2 inhibition.

Therefore, based on the evidence provided in this study, we believe that CLK inhibitors impede angiogenesis by directly targeting the endothelium. However, while this is sufficient in explaining the observed effects, we cannot evaluate effects on the surrounding tissue, especially in the laser-induced CNV model, but also the aortic rings that could contribute to the anti-angiogenic effects resulting from CLK inhibition. Accordingly, we think further research is necessary. Although there is already a small number of studies that have investigated the effects of CLK inhibitors on the release of angiogenic growth factors from non-endothelial cells, available data are still limited and partially contradictory. While the CLK inhibitor KH-CB19 reduced the pro-angiogenic potential of lung cancer cells under hypoxia by altering the expression pattern of tissue factor splice variants [57], inhibiting CLKs in podocytes seems to favor the splicing of VEGF toward pro-angiogenic isoforms [58], which appears to contrast the results from the previous study. Importantly, both studies applied older CLK inhibitors, KH-CB19 and TG003, which are not recommended to be used anymore, because data regarding their selectivity is lacking [15]. Moreover, there are other ways through which CLK inhibitors could influence the release of pro-angiogenic growth factors from the surrounding tissue. For example, our data, together with other publications [14, 15, 18, 51], establish CLK inhibitors as inhibitors of WNT/*β*-catenin signaling, which has been shown to be important for the release of some pro-angiogenic growth factors, like VEGF and IL-8 [59, 60]. Therefore, CLK inhibitors could potentially affect the release of these signaling molecules as well.

Surprisingly, when studying the mechanism of action, we observed that CLK inhibitors do not appear to impact VEGFR2 mRNA splicing, contrary to what one might expect, given the fact that the main known function of CLKs is their involvement in the splicing process [15]. While C81 and MU1210 clearly alter splicing in multiple mRNAs, including CLK1 in accordance with what has been published previously [52], no relevant event upon either treatment was related to VEGFR2. Combined with the fact that C81-derived downregulation of VEGFR2 expression seemed to be largely caused by downregulation of VEGFR2 mRNA expression, we hypothesized that inhibition of a biological process upstream of VEGFR2 mRNA expression is the most likely mechanism. Therefore, we analyzed the downregulated genes in the available RNA-seq data in a GO term analysis and observed that the WNT/*β*-catenin signaling cascade was among the most significantly affected processes. This was interesting to us because it has been comprehensively established that CLK inhibitors impair the WNT/*β*-catenin signaling cascade [14, 18, 51], and because *β*-catenin activity has been shown to affect VEGFR2 expression in HUVECs and retinal endothelial cells [20, 21]. Accordingly, we confirmed that C81 and MU1210 inhibit endothelial WNT/*β*-catenin signaling by utilizing a TCF/LEF reporter gene assay in HMEC-1, using LY2090314 to induce WNT/*β*-catenin signaling through inhibition of glycogen synthase kinase-3 *β* (GSK3*β*) [61]. Furthermore, C81 downregulated the mRNA expression of key WNT/*β*-catenin pathway and target genes, similar to other previously described CLK inhibitors [14, 18, 51], indicating that β-catenin activity was also reduced in HUVECs cultivated without a GSK3*β* inhibitor. Subsequently, we investigated the influence of *β*-catenin activity on VEGFR2 protein and mRNA expression by applying LY2090314 to serum-starved HUVECs. This compound induced VEGFR2 mRNA expression, which was expected based on previous studies on GSK3*β* and *β*-catenin in HUVECs [20, 62], and application of C81 to the LY2090314-treated cells reversed this induction. Conversely, knockdown of *β*-catenin decreased VEGFR2 expression in HUVECs cultivated in fully supplemented ECGM. Therefore, it appears that CLK inhibitors reduce VEGFR2 expression partly by impeding *β*-catenin activity. However, even a strong *β*-catenin knockdown was only able to downregulate VEGFR2 protein expression to about 60%, indicating that CLK inhibitors also affect VEGFR2 expression through other mechanisms, which we plan on investigating in the future.

Moreover, the mechanism through which CLK inhibitors affect WNT signaling remains elusive. It appears from the RNA-seq data that the WNT signaling cascade is more strongly affected by C81 than most other signaling cascades, because it is significantly enriched in a GO term analysis of downregulated genes, indicating selectivity. Previous studies have claimed that CLK inhibitor-derived inhibition of WNT/*β*-catenin signaling is most likely due to induction of alternative splicing in WNT/*β*-catenin-related genes [14, 18, 51]. However, key parts of this hypothesis are still unclear in our opinion. While CLK inhibitors do affect splicing of some WNT-related genes, as described by previous publications and observed in our sequencing data [14, 18, 49, 51], we could not observe any statistical enrichment of WNT/*β*-catenin-related genes in alternatively spliced mRNAs under either treatment with C81 or MU1210. Additionally, the seminal study by Funnel et al., which fully reported all significantly overrepresented signaling pathways in alternatively spliced genes induced by treatment with T3-CLK, did not list the WNT/*β*-catenin pathway as being one of them, despite the fact that great sequencing depth was used [49]. While there is selectivity in inhibiting the pathway activity, this indicates that genes in the WNT/*β*-catenin signaling cascade are not generally more susceptible to alternative splicing induced by CLK inhibition. Therefore, we believe that if induction of alternative splicing is the main reason for CLK inhibitor-derived WNT/*β*-catenin inhibition, there must be a comprehensive mechanism of how (one or more) alternative splicing events cause this to occur. To the best of our knowledge, such an event has not yet been identified. Thus, further investigations should follow.

However, an increasing body of evidence reveals that CLKs are not exclusively important for alternative splicing but also for other cellular processes. It has, for example, been shown that in different cell types, some CLKs are primarily found in the cytosol rather than the nucleus, where splicing takes place [15, 51, 63–65]. Moreover, CLK3 specifically has been shown to activate the ubiquitin-specific peptidase 13 (USP13) through phosphorylation [66], and although they did not further investigate it, coIP-MS experiments published by Funnel et al. showed that the protein most significantly associated with CLK2 was no splicing factor, but USP7 [49]. Accordingly, some of the top hits in the GO term analysis we performed for genes downregulated by C81 treatment were related to ubiquitination as well as other post-translational modifications of proteins. Therefore, we believe that CLKs, at least CLK2 and 3, also play an important role in the ubiquitination and subsequent proteasomal degradation of proteins, which could potentially also play an important role in the mechanism of action of CLK inhibitors. However, these findings are all very new and still largely inconclusive. Thus, investigations into the cellular processes that CLKs are involved in could be a promising new field of research.

In summary, we conclusively show that C81 is an inhibitor of angiogenesis and that other probes for CLKs, as well as CLK knockdowns, mimic its in vitro properties very closely. This, combined with the previously shown fact that CLK inhibitors seem to also reduce inflammatory processes, highlights CLK inhibition as a promising approach in the treatment of diseases that depend on angiogenesis and inflammation, such as arthritis and wAMD. The reduced angiogenic activity is largely due to an inhibition of the VEGF/VEGFR2 signaling cascade, which is mediated by a substantial impairment of VEGFR2 mRNA and subsequent protein expression, all of which is a consequence of CLK inhibition, as CLK knockdown phenocopy reduced angiogenic activity and VEGFR2 mRNA expression in HUVECs. Mechanistically, impeded WNT/*β*-catenin activity seems to be partly responsible for the observed effects, but cannot fully explain them, whereas induction of alternative splicing in VEGFR2 mRNA does not appear to occur. However, more research is sorely needed to elucidate how VEGFR2 is downregulated by CLK inhibitors beyond WNT/*β*-catenin inhibition, and, more importantly, what mechanisms are responsible for the observed impairment of WNT/*β*-catenin signaling derived from CLK inhibition.

### Supplementary Information

Below is the link to the electronic supplementary material.

Supplementary material 1 (PDF 7719.1 kb)

Supplementary material 2 (XLSX 10.0 kb)

Supplementary material 3 (CSV 30.5 kb)

Supplementary material 4 (CSV 95.0 kb)

Supplementary material 5 (XLSX 8.8 kb)
