# Supplementary material for: 2-Desaza-annomontine (C81) impedes angiogenesis through reduced VEGFR2 expression derived from inhibition of CDC2-like kinases
Source: Angiogenesis. 2024 Feb 26;27(2):245–72. doi: 10.1007/s10456-024-09906-y (PMC11021337; doi:10.1007/s10456-024-09906-y)
Supplement: Supplementary file 1 — Supplementary material 1 (PDF 7719.1 kb) [file 10456_2024_9906_MOESM1_ESM.pdf]

Supplementary figures to:

2-Desaza-annomontine impedes angiogenesis through reduced VEGFR2 expression derived from inhibition of CDC2-like kinases.

Zech, T. J.[1]; Wolf, A.[2,3]; Hector, M.[2]; Bischoff-Kont, I[1].; Krishnathas, G. M.[1]; Kuntschar, S.[4]; Schmid, T.[4]; Bracher, F.[5]; Langmann, T.[2,3]; Fürst, R.[1,6]

[1] Institute for Pharmaceutical Biology, Faculty of Biochemistry, Chemistry and Pharmacy, Goethe-University of Frankfurt, Frankfurt, Germany

[2] Laboratory for Experimental Immunology of the Eye, Department of Ophthalmology, University of Cologne, Faculty of Medicine and University Hospital Cologne, Cologne, Germany

[3] Centre for Molecular Medicine Cologne (CMMC), University of Cologne, Cologne, Germany

[4] Institute of Biochemistry I, Faculty of Medicine, Goethe-University of Frankfurt, Frankfurt, Germany

[5] Pharmaceutical Chemistry, Department of Pharmacy – Center for Drug Research, Ludwig-Maximilians-Universität München, Munich, Germany

[6] Pharmaceutical Biology, Department of Pharmacy – Center for Drug Research, Ludwig-Maximilians-Universität München, Munich, Germany

For correspondence, please contact Prof. Robert Fürst, Pharmaceutical Biology, Department of Pharmacy – Center for Drug Research, Ludwig-Maximilians-Universität München, Munich, Germany
Robert.Fuerst@cup.uni-muenchen.de

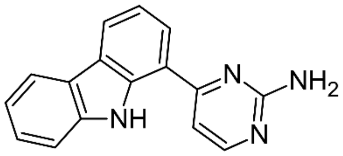

C81

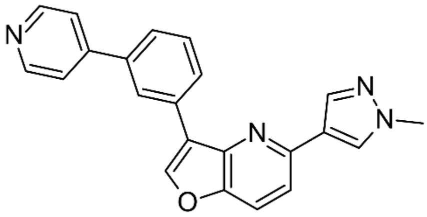

MU1210

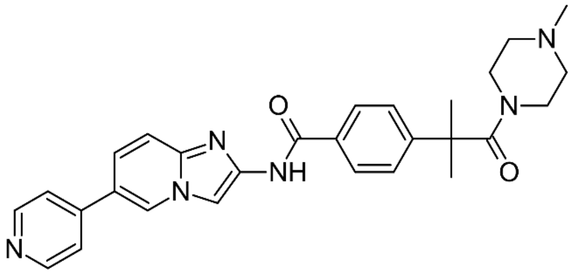

T3-CLK

Supplementary Fig. 1 Structures of C81, MU1210 and T3 CLK
Structures were drawn using ChemSketch (ACD/Labs).

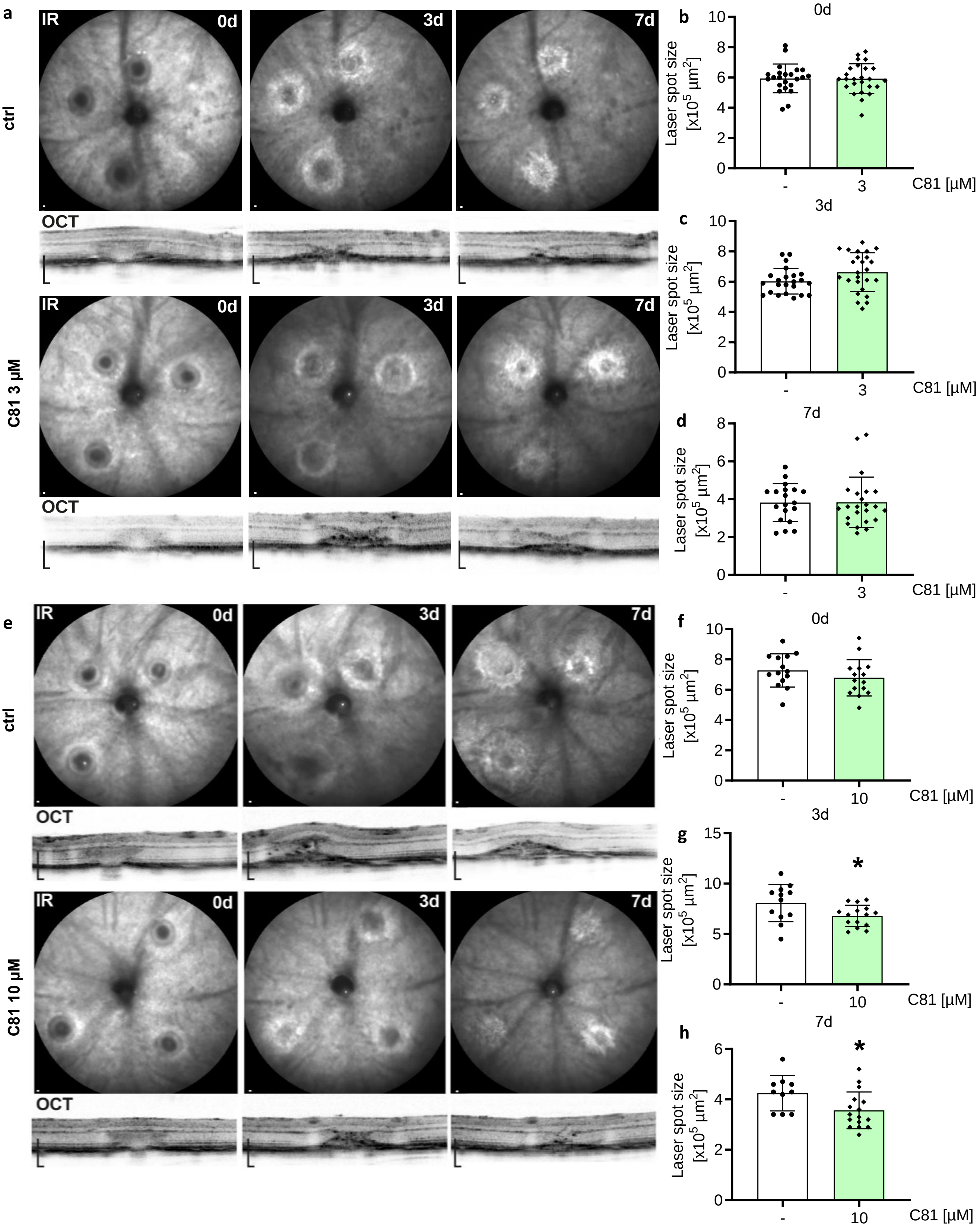

Supplementary Fig. 2 Laser spot size in CNV model

a/e Representative infrared (IR) fundus images at indicated time points and treatments post laser injury. Lower panel shows OCT scan from one laser spot. Scale bar: 200 µm. **b-d/f-h** Quantification of laser spot size. **b-d** n = 19-25 eyes, **f-h** n = 10-15 eyes. Data show mean ± SD, two-tailed unpaired Student's t test; $^{*}p \leq 0.05$

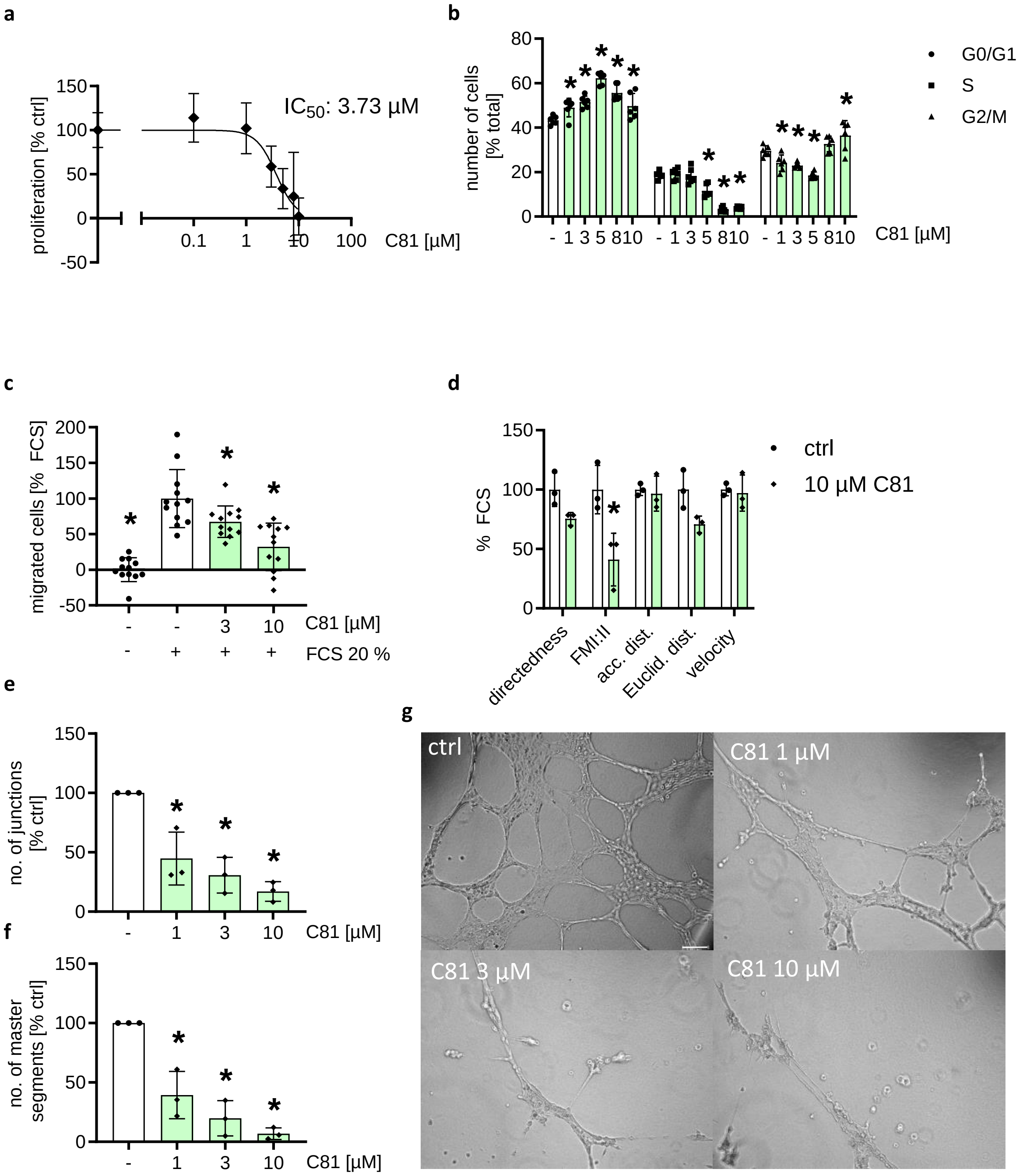

Supplementary Fig. 3 Effect of C81 on proliferation chemotactic migration and tube formation of HUVECs
a Quantification of proliferation of HUVECs in a crystal violet assay. **b** Distribution of HUVECs in the indicated stages of the cell cycle quantified through Propidium iodide staining and Flow cytometry. **c** quantification of migration of HUVECs along a 0-20 % FCS gradient by crystal violet staining. **d** Quantification of directedness, forward migration index in parallel to the chemoattractant, accumulated distance, Euclidian distance, and velocity of scarcely seeded HUVECs along a 0-20 % FCS gradient over 20 h. **e/f** Quantification of tube formation for number of junctions **(e)** or number of master segments **(f)**. **g** representative images of HUVEC tubes on Matrigel. **a-d/e/f** Data show mean ± SD, **a** IC50 was calculated using a nonlinear regression with a variable slope **b/d** two-way ANOVA with Dunnett's post hoc test testing for a simple effect within cell cycle stages **(b)** or mode of quantification **(d)**. **c/e/f** One-way ANOVA with Dunnett's post hoc analysis comparing treatments and no-gradient control vs vehicle control **(c)** or comparing treatments to vehicle control **(e/f)**. *$p \leq 0.05$ vs vehicle control within cell cycle stage **(b)**, vehicle control **(c/e/f)** or mode of quantification **(d). g** Scalebar represents 100 µm.

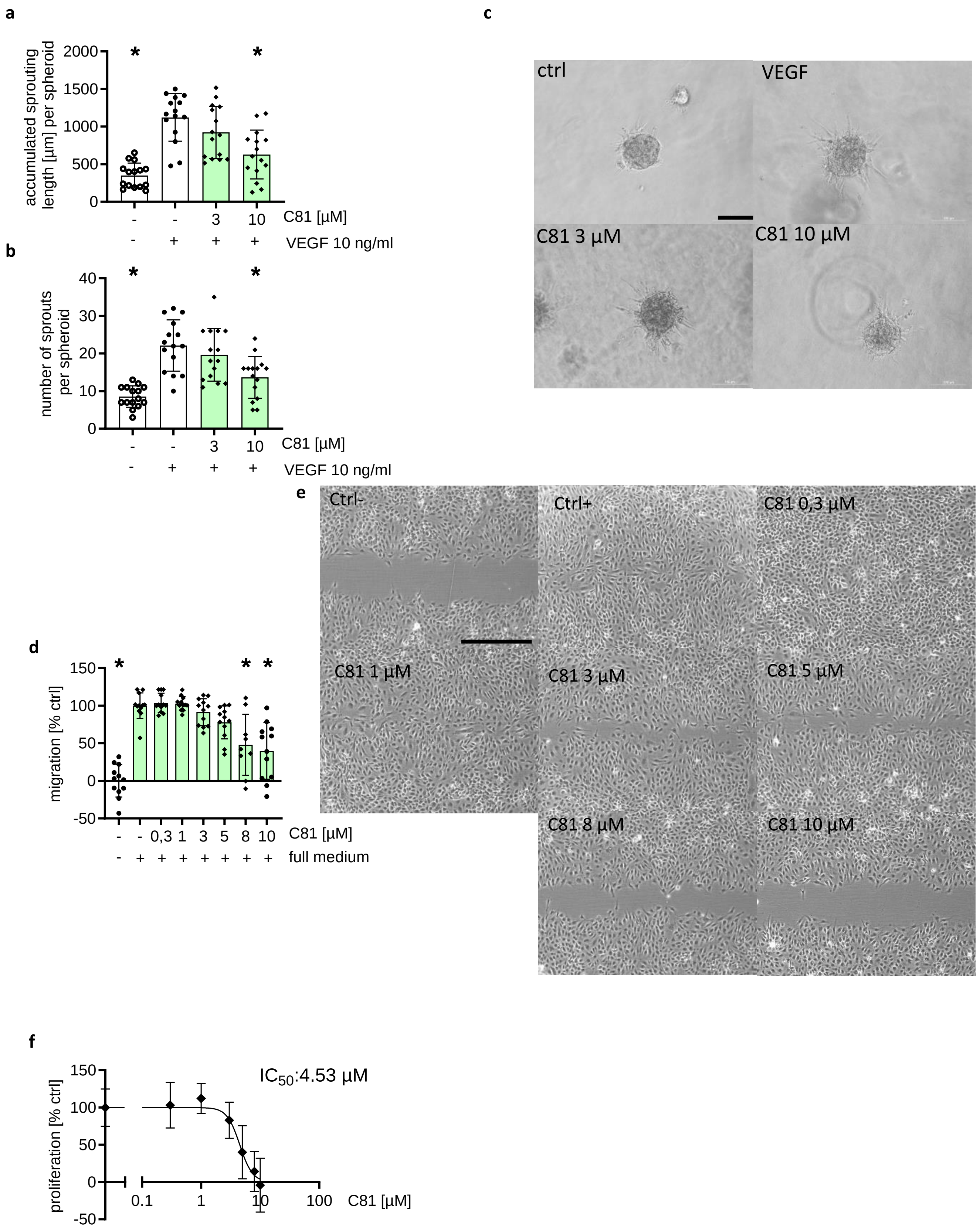

Supplementary Fig. 4 C81 inhibits angiogenic key steps *in vitro* in HMEC-1

a/b Quantification of HMEC-1 spheroids pretreated for 30 min with the indicated concentrations of C81/vehicle control and stimulated with VEGF for 20 h for accumulated sprouting length per spheroid **(a)** and number of sprouts per spheroid **(b)**. **c** Representative images of collagen embedded HMEC-1 spheroids at the end of treatment. **d** Quantification of scratches for closed surface area under treatment of C81, negative ctrl served as a baseline. **e** Representative images of scratches after incubation. **f** Quantification of proliferation of HMEC-1 in a crystal violet assay

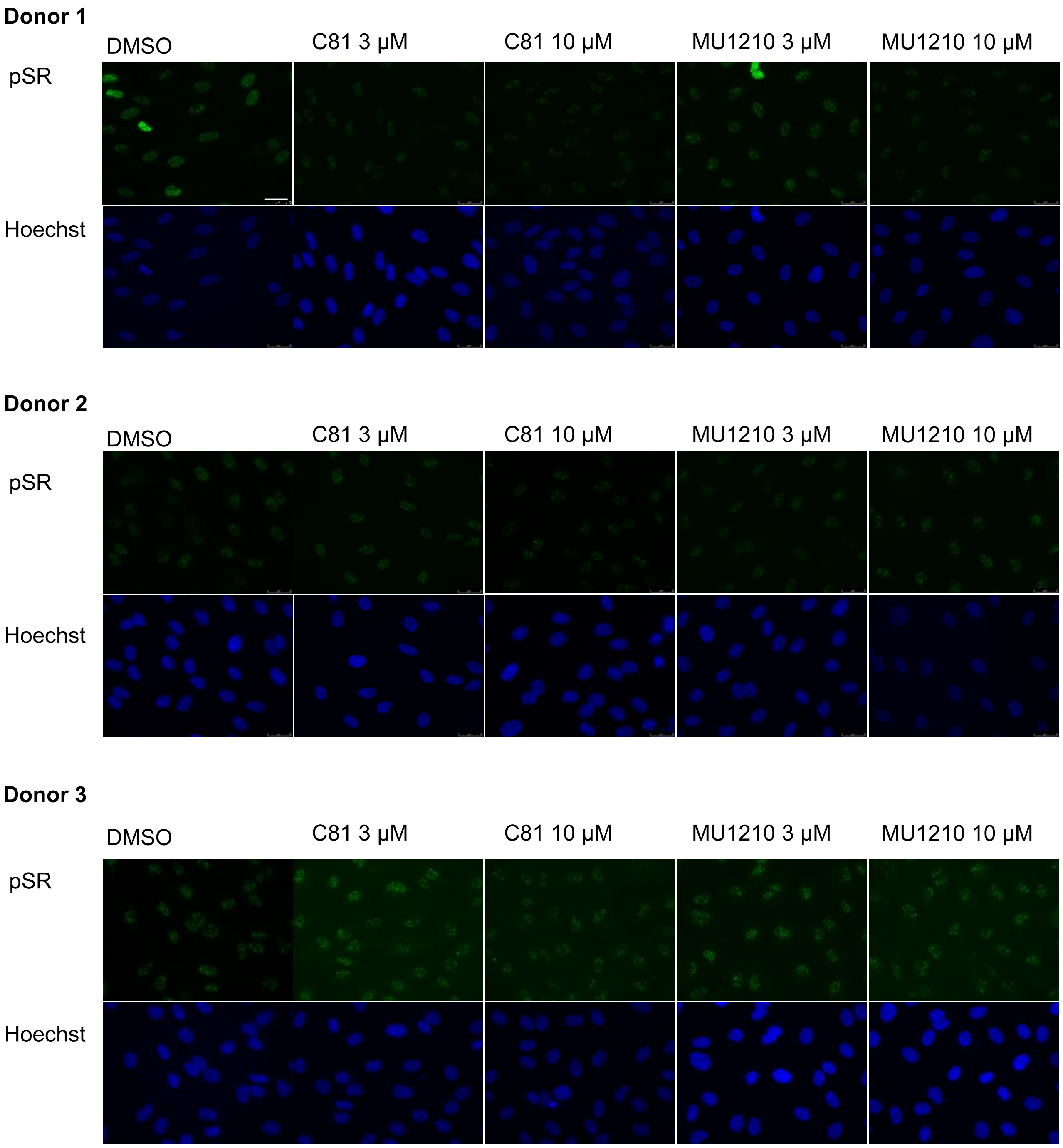

Supplementary Fig. 5 Unedited images of IGCs in HUVECs
Immunofluorescence pictures of HUVECs stained with Hoechst 33342 for the nuclei and anti-p-SR proteins to mark interchromatin granule clusters. Altered distribution can be observed when HUVECs are treated with C81 or MU1210 for 6 h.

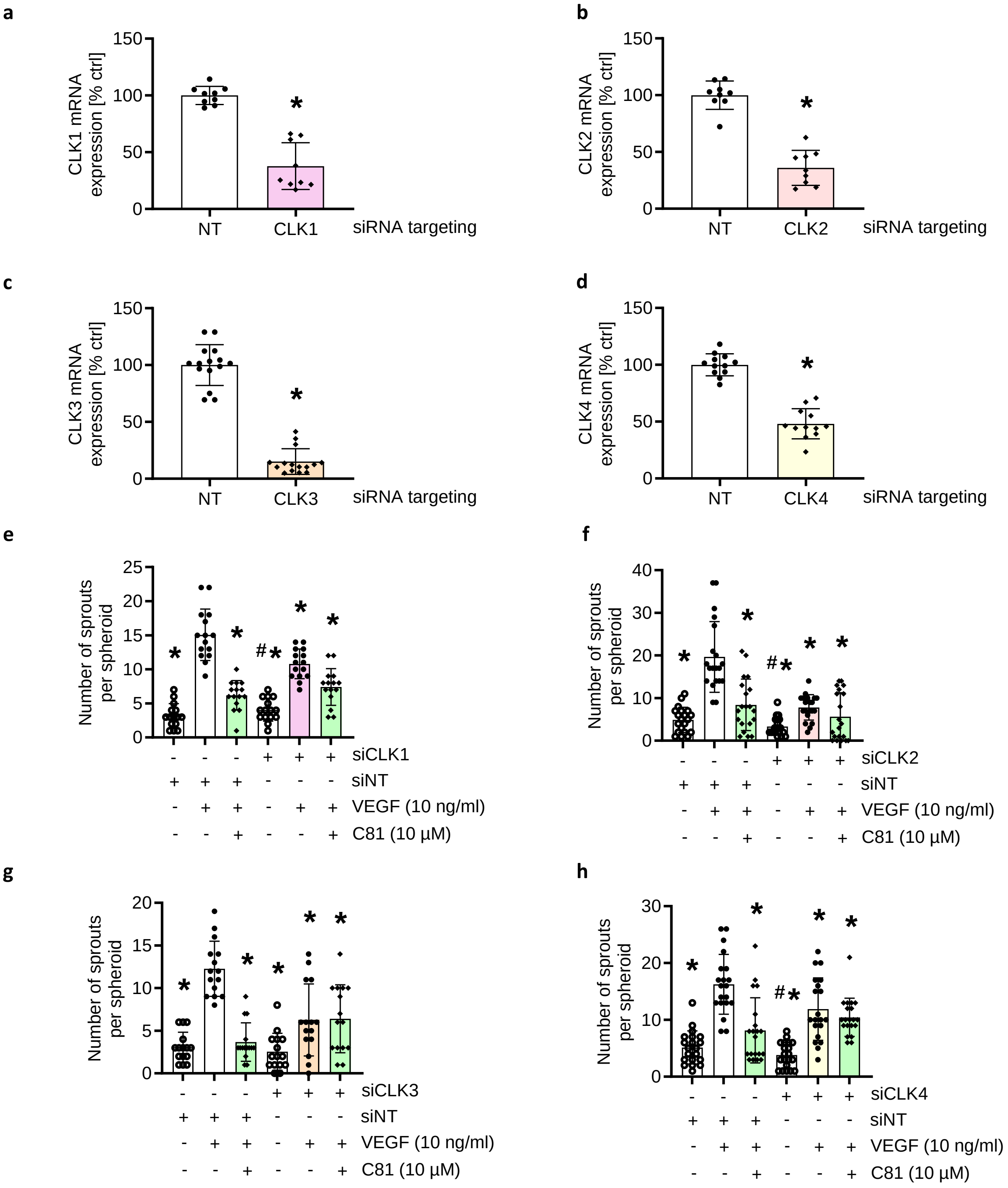

Supplementary Fig. 6 Quantification of CLK1-4 knockdowns and additional quantification of knockdown spheroids

a-d Relative quantification of CLK1-4 mRNA expression, normalized to GAPDH, 72 h after the indicated knockdown was induced in HUVECs. **e-h** Quantification of HUVEC spheroids embedded into collagen 72 h after the indicated knockdown was induced, pretreated for 30 min with the indicated concentrations of C81/vehicle control and stimulated with VEGF for 20 h for number of sprouts per spheroid. **a-h** Data are represented as mean ± SD, n = 3 donors **(a-e/g)** or n = 4 donors **(f/h). a-d** unpaired student's t-test; **e-h** one-way ANOVA with Tuckey's post hoc test. **a-h** *p ≤ 0.05 compared to non-targeting control **(a-d)** or VEGF non targeting control **(e-h)**; **e-h** #p ≤ 0.05 compared to knockdown cells stimulated with VEGF, only used to compare treatments of knockdown cells.

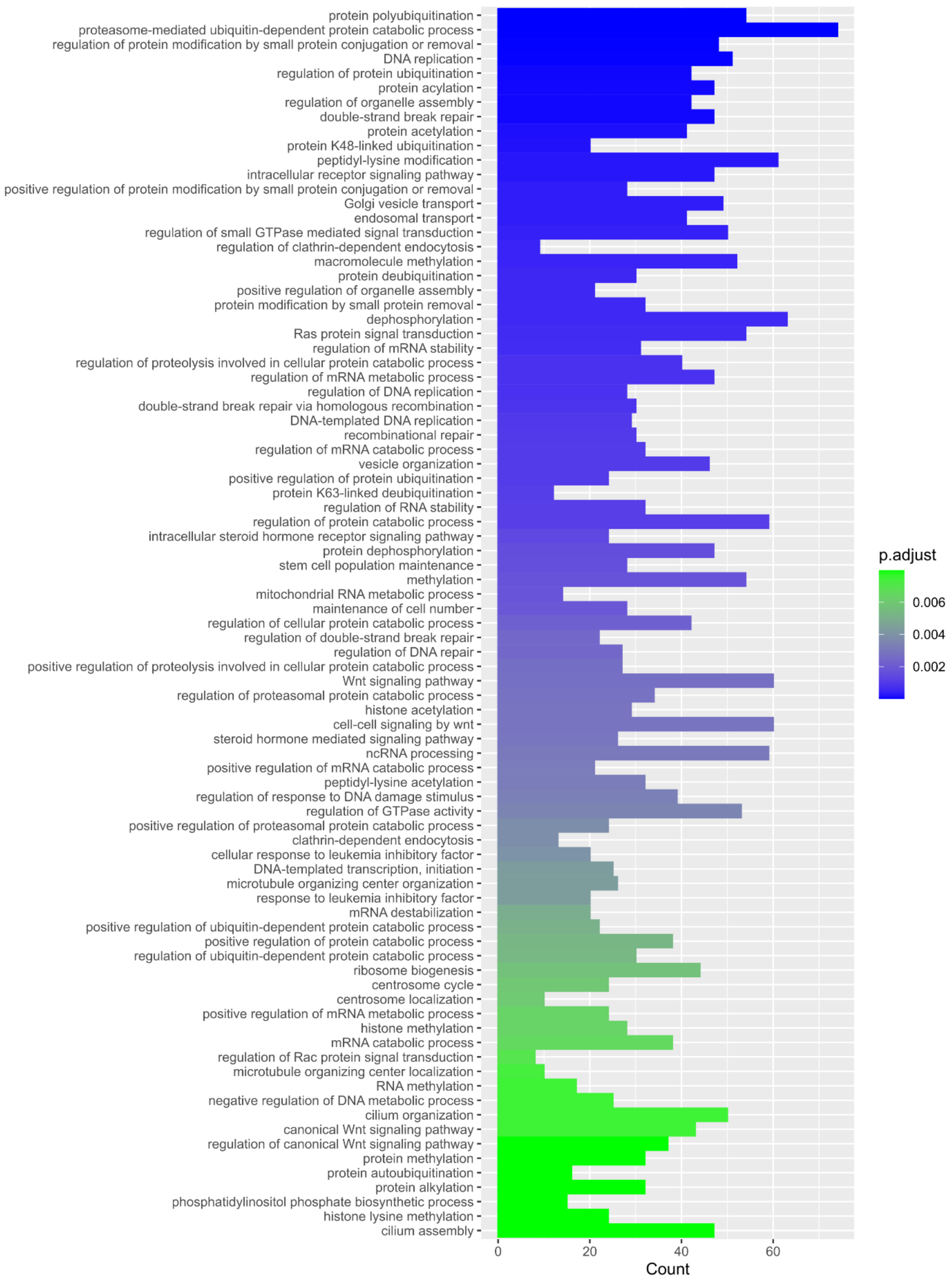

Supplementary Fig. 7 RNA-Seq of C81 treated cells detects multiple affected biological processes, and notably WNT/β-catenin as an affected signaling pathway

GO-Term analysis of biological processes affected by 6 h treatment of HUVECs with 10 µM C81 compared to vehicle control, detected using short read RNA-Seq. N = 3 donors.

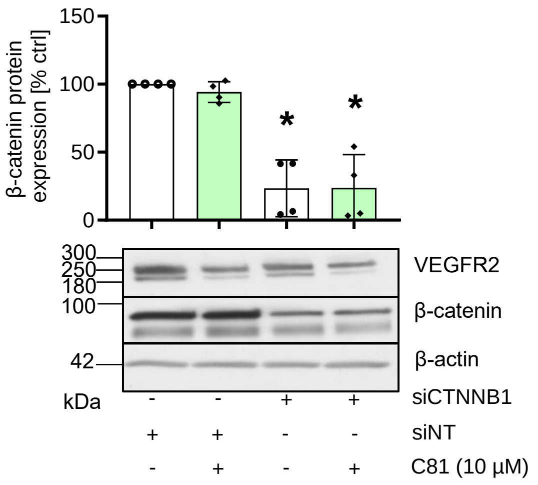

Supplementary Fig. 8 β-Catenin knockdown efficiency

Semiquantitative analysis of β-catenin protein expression, normalized to β-actin, using densitometry in Fiji/ImageJ in HUVECs 48 h after knockdown was induced. C81 was added for the final 10 h of the incubation. Data are depicted as mean ± SD, one-way ANOVA with Dunnett's post hoc test, *$p \leq 0.05$ compared to the non-targeting control.
